# Phylogeography of *Pteronotropis signipinnis*, *P. euryzonus*, and the *P. hypselopterus* Complex (Teleostei: Cypriniformes), with Comments on Diversity and History of the Gulf and Atlantic Coastal Streams

**DOI:** 10.1155/2015/675260

**Published:** 2015-05-31

**Authors:** Richard L. Mayden, Jason Allen

**Affiliations:** ^1^Department of Biology, Saint Louis University, 3507 Laclede Avenue, St. Louis, MO 63103, USA; ^2^Department of Biology, Saint Louis Community College-Meramec, 11333 Big Bend Road, St. Louis, MO 63122, USA

## Abstract

The cyprinid genus* Pteronotropis* is endemic to southeastern Gulf of Mexico and Atlantic Ocean of North America. Never before has the genus been demonstrated to be monophyletic. We investigate both the phylogenetic relationships and the phylogeography of some species in the genus using mitochondrial ND2 sequences. In no analysis is the genus resolved as monophyletic if* Notropis harperi* is not included in the genus. Biogeographic and phylogeographic evaluations are conducted with* Pteronotropis*, including* P. signipinnis*,* P. euryzonus*, and the* P. hypselopterus* complex. Patterns of relationships and population genetic analyses support divergences within multiple clades both at the species level and within species that are tied to abiotic changes in the region. Replicated patterns across clades are observed, as well as patterns previously found in other taxa. Pteronotropis hypselopterus is likely not a natural grouping as populations from some drainages form clades more closely related to other species of the genus. The general patterns of relationships indicate likely cryptic species not currently recognized. Finally, the patterns of species relationships and clades and population structuring within species serve as another example of replicated divergences in the biodiversity east and west of the Mobile Bay.

## 1. Introduction

Avise [1] defines phylogeography as “… a field of study concerned with the principles and processes governing the geographic distributions of genealogical lineages, especially those within and among closely related species.” Phylogeography is a subdiscipline of historical biogeography, which seeks to find historical explanations for the present distribution of organisms [2]. Within this framework, two competing hypotheses have existed for over a century to explain how species and their populations came to occupy a geographic area or aquatic system, dispersal and vicariance. Dispersalists favor the hypothesis that the present distributions of organisms are explained by movement of populations; closely related taxa separated by some type of barrier significant to them diverged once some populations were successful in overcoming this barrier and were isolated long enough to diverge from their sister group. Vicariant biogeographers seek to explain the distribution of related taxa by hypothesizing that part of the geographic range of an ancestral species became fragmented by some barrier, isolating some populations that later diverged. The hypothesis of dispersal in the past is one that is largely impossible to test. The theory behind vicariance biogeography stipulates that vicariant patterns should be used as a first-order explanation for the distribution of organisms and only if this hypothesis is rejected should dispersal be invoked. Thus, vicariance biogeography does not stipulate that dispersal does not occur. Further, this model of divergence maintains that if a variety of taxa show concordant patterns around the same barrier then the vicariant event underlying the diversification event across lineages is the most parsimonious explanation. The confidence that we have in these concordant patterns is really a function of how often empirical evidence corroborates their occurrence. Fishes from rivers and streams of the southeastern United States along the Gulf and Atlantic slopes have been the attention of varied phylogeographic studies, including those by Wiley and Mayden [3], Swift et al. [4], Nagle and Simons [5], and Sandel [6] as well as studies referenced therein.

**Figure 1:**
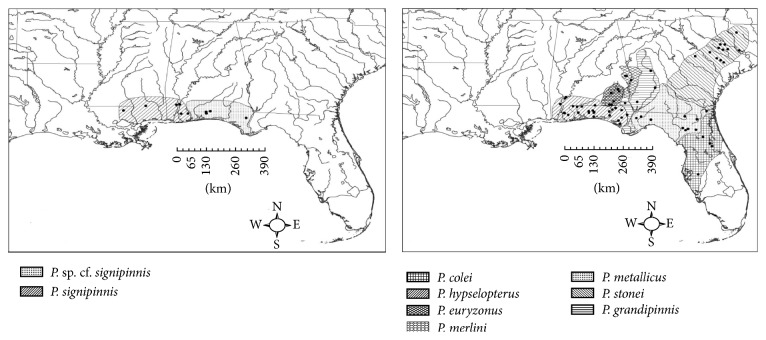
Distributions and sampling localities for* Pteronotropis signipinnis*,* P. euryzonus*, and the* P. hypselopterus* complex.

The cyprinid genus* Pteronotropis* is largely endemic to aquatic habitats of the southeastern United States and contains species with small and large distributions across this large geographic area (Figure 1). The genus was reviewed by Suttkus and Mettee [7] and Mayden and Allen [8]. Not until the latter was the genus corroborated as a monophyletic with two major lineages [8]. The distributions of species of* Pteronotropis*, combined with the many rivers systems that they occupy and independently enter the Gulf of Mexico or Atlantic Ocean, make species of the genus of particular interest for both systematic and biogeographic evaluations. The geological processes and timing of both geological and hydrological events across this region are relatively well known, the lower reaches of rivers have been inundated by fluctuating sea levels, and multiple species inhabit coastal areas [4]. The species diversity of* Pteronotropis*, distributions of species, and the history of the area provide for an attractive model to investigate their historical biogeography and compare to other species inhabiting this same area. Herein, we examine the evolutionary and biogeographic history of* Pteronotropis* using both sequences of one of the most appropriate genes for the scale of diversity being examined, ND2. We use this genetic information and appropriate statistical tests to explore hypotheses of the evolutionary history of targeted species.

Statistical parsimony, although technically a phenetic method (based upon overall similarity and not shared derived characters as in phylogenetics), can offer valuable insight into relationships of closely related individuals (i.e., populations within a single species) where the resolution of true parsimony may fail. Functionally, the method converts the haplotype tree of each species into a series of networks showing differences to the level of single mutational events [9]. This technique is widely accepted as a means of exploring phylogeographic relationships and has been used in many studies of Nearctic and Palearctic fishes [10, 11]. Mismatch distribution analysis (MDA) essentially takes the distribution of pairwise differences (mismatch distributions) calculated earlier and plots them against the number of individuals in the analysis [12]. The advantage of this method is its ability to distinguish between rapid range expansion and expansion through recurrent gene flow. If rapid range expansion has occurred in the history of a species, the expectation is that the plot will show a unimodal distribution of pairwise differences; a multimodal distribution would indicate population stability [13]. This technique was used in many demographic studies, including those of human [14] and fish populations [15]. Mitochondrial genes are very useful as phylogeographic markers because of their generally fast rate of anagenesis, uniparental mode of inheritance, and lack of recombination, all allowing researchers to detect events occurring at the population level and track events over geographical ranges and through evolutionary time [1]. In particular, ND2 is an effective marker in distinguishing relationships at many levels [16]. Two nuclear genes, the third exon of RAG 1 and the first intron of S7, used in Mayden and Allen [8], were tested for use in this analysis but lacked sufficient variation to be informative for the questions being examined herein; likewise, Chen and Mayden [17] and Mayden and Chen [18] examined variation in an additional six nuclear genes, but these genes also provided insufficient variation to test hypotheses of inter- and intraspecific relationships at this level. Finally, the southeast as a whole has served as a paradigm for phylogeographic research with many of its principles and techniques being developed from studies on taxa endemic to this region. Consequently, many congruent patterns have emerged for numerous species ranging from freshwater fishes, amphibians, reptiles, mammals, macroinvertebrates, plants, and maritime species [1, 3, 19, 20]. Species and species complexes of* Pteronotropis* are also endemic to streams of the southeastern United States. Furthermore, the genus* Pteronotropis* has never been well corroborated as monophyletic. A previous analysis of the group by Suttkus and Mettee [7] developed specific hypotheses as to the diversification and biogeography of the group. Thus, this species complex serves as another excellent candidate group to examine the historical patterns of biodiversity along the Atlantic-Gulf slopes and to test multiple hypotheses as to dispersal, vicariance, speciation, and divergence of populations.

## 2. Review of the Geography and Diversity of the Region

Swift et al. [4] define the southeastern United States as areas occurring south, southeast, and southwest of the peripheral Tennessee River system, west to Lake Pontchartrain and east to the Savannah River. This includes aquatic systems from the entire states of Alabama, Florida, Georgia, Mississippi, and part of South Carolina. Geologically the southeast can be thought of as three separate areas: (1) a mountainous area consisting of Paleozoic and Mesozoic rocks, (2) a lower relief hilly terrain made up of pre-Cretaceous formations termed the Piedmont, and (3) a sedimentary terrain of very low relief termed the Coastal Plain [21]. The region separating the Piedmont from the Coastal Plain is the “fall line” and often forms the distributional boundary between many upland and lowland species. The southeast has been a model of geographic stability since the middle to late Cretaceous period with no major land movements or continental shifting occurring since this time [22]. This is also thought to be true of the river systems, which traverse this region, with most of the drainage basins being in place since the late Cretaceous to early Eocene. The southeast includes about 32 major to minor river systems. These generally have a pattern of flow to the southeast or southwest, depending on where they discharge, with the larger drainage systems having their headwaters above the fall line. The larger river systems include the Pearl, Pascagoula, Tombigbee (including Black Warrior River), Alabama (including Cahaba, Coosa, and Tallapoosa rivers), Chattahoochee, Altamaha, and Savannah rivers. The remaining rivers are much smaller in their drainage basins, have their headwaters originating below the fall line, and include the Escambia, Choctawhatchee, Ochlocknee, Suwannee, St. Marys, and Satilla Rivers [4]. Rivers originating at or above the fall line tend to have high gradient and a substrate mainly consisting of bedrock, boulders, and gravel. Streams below the fall line are typically lower gradient and have slower flows and substrates largely consisting of clay or sand, with some gravel or bedrock in some areas. These streams typically flow through forested swamps and wetlands, and it is because of this that they get their name “Blackwater streams” and are typically described as being “tannin stained,” from the leaching of tannins from decaying vegetation. Even though the land formations of this region have been stable over millions of years, sea level fluctuations have been an impact on the region and proposed to have had a major impact on the biodiversity of this region [4].

It is estimated from fossil record that towards the end of the Oligocene sea levels fell dramatically and exposed a large continental shelf due to the development of polar ice caps and the filling of the Mediterranean Sea [4]. These events were followed by an elevation of sea level to about 100 meters above present levels in the mid to early Miocene. A slight drop in water level occurred again at the end of the Miocene followed by another rise in the early Pliocene, with slight fluctuations during the Pleistocene glaciations. During the high sea stands the smaller rivers below the fall line were likely completely obliterated, leaving obligate freshwater taxa to inhabit the refugia of isolated upstream sections in the larger rivers. As waters receded it is hypothesized that suitable habitat became available in lower reaches of the larger rivers coinciding with the appearance of smaller streams and rivers. Although no drainage connections exist between most of these rivers today, during periods of low sea stands there were coalescent events between many of these streams, enabling taxa to disperse throughout these systems [5, 6]. This history likely explains why many of the smaller rivers below the fall line, such as the Escambia, Blackwater, and Yellow, have no modern-day connections, yet contain most of the same taxa with few, if any, endemics [4].

One of the classical concordant patterns of diversification in this region has been the genetic distinction between Atlantic and Gulf slope taxa, with the Apalachicola drainage system being the range limit or contact zone for taxa on either side. One of the first studies to show this was that of Bermingham and Avise [19], who in their comparison of four freshwater fish species (*Amia calva*,* Lepomis punctatus*,* L. microlophus*, and* L. gulosus*) observed significantly greater genetic differentiation between Atlantic and Gulf slope populations of each species than that observed among haplotypes within each region. These patterns of diversification for freshwater fishes have been confirmed by Swift et al. [4], in their study of the zoogeography of the southeast, using a simple present-absence matrix for almost all the freshwater species and illustrated phenetically that the “oldest” split was between the Gulf Slope streams, up to and including the Apalachicola, and southeastern Atlantic Slope streams, including all of those of Florida.

Another often cited pattern in the region is that associated with the Central Gulf slope speciation hypothesis proposed by Wiley and Mayden [3]. In their work on vicariant patterns in the North American freshwater fish fauna they identified several sister species and populations within a single species that have their distributional limits defined by the Mobile Basin. Some of these taxa include the* Fundulus nottii* species group,* Ammocrypta beani* and* A. bifasciata*,* Etheostoma chlorosomum* and* E. davisoni*, and populations of* Notropis longirostris*. In a study on the* Notropis dorsalis* species group, using the mitochondrial marker cytochrome* b*, Raley and Wood [23] showed that populations of* N. longirostris* on either side of the Mobile Basin were resolved as two separate clades with as much as 8% sequence divergence. Swift et al. [4] provide a possible mechanism for these occurrences by hypothesizing that the Alabama-Tombigbee river system had more of a westward or southwestward flow pattern in the early Miocene that was diverted directly southward during the middle to late Miocene, thus dividing populations of species on either side of this river system.

The* Pteronotropis hypselopterus* species complex occurs in streams extending across the Atlantic-Gulf slopes (Figure 1).* Pteronotropis hypselopterus* occurs from western tributaries of the Mobile Bay eastward to, but not including the Apalachicola River drainage.* Pteronotropis merlini* is endemic to the Choctawhatchee River system, including the Pea River, at and above the confluence of the east and west forks. Any* Pteronotropis* below this confluence is considered to be* P. hypselopterus* [7].* Pteronotropis grandipinnis* is endemic to the Apalachicola River system in the lower reaches of the Chattahoochee and Flint rivers.* Pteronotropis euryzonus*, though not part of the* P. hypselopterus* complex, is closely related to this group [8] and is endemic to the middle Chattahoochee River. The two remaining species are endemic to Atlantic slope rivers and include* P. metallicus,* ranging from the Ochlocknee River to the St. Johns and Hillsborough rivers of peninsular Florida, and* P. stonei* with its northern distributional limit in the lower reaches of the Pee Dee River, South Carolina, to as far south as the Satilla River in southern Georgia.

Suttkus and Mettee [7] offered the most current biogeographical account for members of this genus. They contended that* P. euryzonus* evolved below the fall line in the middle Chattahoochee River system, where still endemic, and then spread to the adjacent Choctawhatchee River system through a temporary stream capture. These ancestral populations were then dispersed throughout the Choctawhatchee and Pea river systems, eventually giving rise to* P. merlini* above the confluence of these two rivers and* P. hypselopterus* below, possibly through a vicariant event such as habitat specialization.* Pteronotropis merlini* is thought to be more of an upland species and* P. hypselopterus* a more swampy lowland species. Thus, under the hypothesis of Suttkus and Mettee [7]* P. hypselopterus* populations then migrated as far west as the Mobile Bay area and east to the Apalachicola River Drainage, and via a stream capture with the Ochlocknee River, it dispersed further northeast.

As further described by Suttkus and Mettee [7] populations of* Pteronotropis hypselopterus* in the Apalachicola River Drainage eventually became isolated from other populations expanding to the north and east and as ancestral populations eventually gave rise to* P. grandipinnis*. Eventually, the* P. hypselopterus* stock spreading east gained access to the Suwannee and St. Mary's rivers and through interconnecting drainages spread as far north as the Pee Dee River System in South Carolina and as far south as the Myakka River in peninsular Florida. Through changes in drainage patterns the once continuous population of* P. hypselopterus* ranging from South Carolina to Florida became fragmented with the South Carolina and Georgia populations evolving into* P. stonei* and the Florida populations east of the Apalachicola diverging into* P. metallicus* [24].

## 3. Methods

For the phylogeographic analysis, multiple populations and multiple individuals throughout the ranges of species of the* P. hypselopterus* complex, as well as* P. signipinnis*,* P. euryzonus*,* P. hubbsi*,* P. welaka*, and* P. harperi*, were sequenced for the mitochondrial gene ND2.* Pteronotropis harperi* is included in this analysis as almost all previous analyses of* Pteronotropis* [8, 25–28], using both mitochondrial (Cytochrome* b*, 12S and 16S ribosomal RNA) and nuclear (RAG 1 and S7) sequences, have corroborated the hypothesis that this species is imbedded within* Pteronotropis*. Included in our analyses were* P. hypselopterus* (*n* = 65; 25 localities),* P. merlini* (*n* = 10; 4 localities),* P. grandipinnis* (*n* = 9; 4 localities),* P. metallicus* (*n* = 31; 10 localities),* P. stonei* (*n* = 21; 11 localities),* P. signipinnis* (*n* = 23; 11 localities),* P. euryzonus* (*n* = 8 individuals; 3 localities),* P. welaka* (*n* = 5; 4 localities),* P. hubbsi* (*n* = 4; 2 localities), and* P. harperi* (*n* = 12; 5 localities) for a total number of 188 individuals from 79 localities. Taxa purported earlier to be the close relatives of* Pteronotropis* were used as outgroups and included species of* Notropis*,* Cyprinella*, and* Lythrurus* (based on previous classification of* Pteronotropis* and these three genera previously in* Notropis* [29, 30]).* Pteronotropis welaka* and* P. hubbsi* also served as outgroups based on phylogenetic reconstructions by Mayden and Allen [8, 25–28]. A complete listing of sample records is provided in Table 1.

Complete genomic extractions were performed using QIAgen QIAamp tissue kits (QIAGEN, Valencia CA). The entire mitochondrial ND2 coding region was amplified using PCR with the following conditions: denaturation 94°C for 40 seconds, annealing 56°C for 60 seconds, and extension 72°C for 90 seconds. This was performed for 35 cycles with each 50 L PCR reaction consisting of 4 L of DNTPs, 5 L of 10X* Taq* buffer, 2.5 L of both forward and reverse primers, 30.7 L of dH_2_O, 5 L of MgCl_2_, and 0.3 L of* Taq.* PCR product purification was performed using either a QIAgen gel extraction kit (QIAgen, Valencia CA) or an Agencourt AMPure purification kit (Agencourt Biosciences, Beverly MA). Sequencing was performed using a big dye labeled dideoxy sequencing kit (Big Dye) and visualized on an ABI 377 automated sequencer (Auburn University Molecular Genetics Instrumentation Facility, Auburn, AL) or an ABI 3700 (Macrogen Sequencing Facility, Seoul, South Korea). Sequences were edited and aligned by eye using BioEdit versus 0.9 (Hall [31]).

Parsimony analyses were initially run in PAUPrat [32] using 5–25% character permutations. The best tree found from these analyses was used in all subsequent parsimony analyses. Maximum parsimony (MP; MPA = MP analysis) was performed in PAUP^*∗*^ [33] with 1000 random addition sequence replicates and tree bisection-reconnection branch swapping (TBR). All characters were equally weighted and unordered. Likelihood analyses were performed using the general algorithm for rapid likelihood inference (GARALI) [34, 35] and the GTR+ G+I model of evolutionary change; the tree with the best likelihood score was retained and is presented as the optimum ML topology. Bootstrap analysis (BA) was completed using 100 bootstrap pseudoreplicates [36]. Bayesian analyses used MrBayes [37] with four heated Markov chains and default temperature setting. Each analysis was run for 1 million generations with sampling every 250 generations. Log-likelihood scores were plotted against generation time to establish burn-in; trees before stationary were discarded. A 50% majority rule consensus tree was generated from the remaining trees.

Uncorrected and corrected genetic distances were calculated using MEGA 3 [36]. Statistical parsimony [38, 39], as implemented in TCS 1.18 [40, 41], was used to group haplotypes into a minimum-connecting networks to illustrate potential genealogical connections. Mismatch distributions of the number of differences between haplotypes [12] and genetic statistics were conducted using DnaSP version 3 [42] to examine demographic differences between clades within the trees.

## 4. Results

Sequences for the complete ND2 gene (1047 bp) yielded 172 haplotypes from among 188 individuals from 79 localities across the range of* Pteronotropis*. Of the 1047 characters identified, 517 were parsimony informative (49.5%). MPA resulted in 1867 equally parsimonious trees with 2437 steps (CI = 0.452, RI = 0.654). ML analyses resulted in a most likely tree log value of −41397.64. Bayesian analyses reached stationary after 100,000 generations; trees from the first 125,000 generations were discarded as burn-in, leaving 11,764 trees for phylogeny estimation.

**Figure 2:**
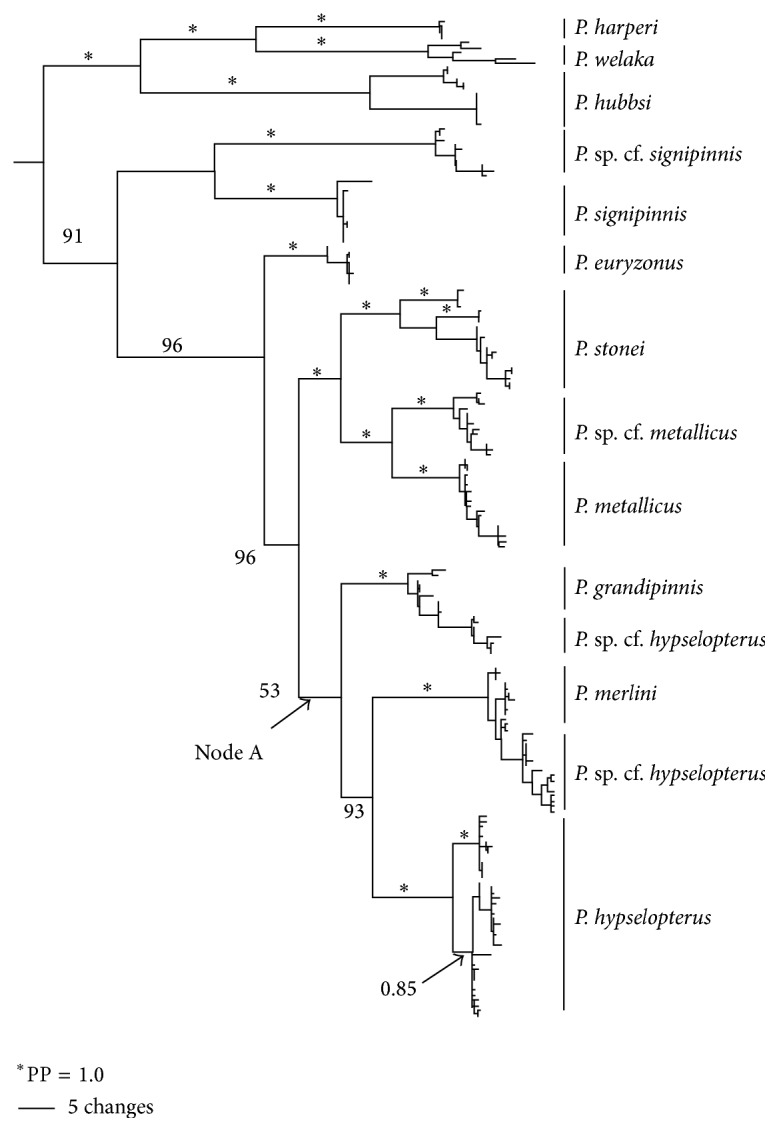
Relationships of species and populations of* Pteronotropis* as inferred from Bayesian analysis of ND2 gene sequences and summarized using a 50% majority rule consensus tree. *∗* = 100PP.

MP, ML, and BA all recovered 10 strongly supported major clades within* Pteronotropis* with essentially identical topologies; only Bayesian trees are illustrated for further discussion. ND2 sequences failed to resolve* Pteronotropis* as a monophyletic group without the inclusion of* Notropis harperi*, a species now included in* Pteronotropis* by Mayden and Allen [8] from their analyses using two nuclear genes. The overall topology of the tree (Figure 2) reveals two reciprocally monophyletic groups, the* Pteronotropis harperi* clade sister to the* Pteronotropis signipinnis* clade. The* P. harperi* clade includes* P. welaka*,* P. hubbsi,* and* P. harperi*. Within this group* P. hubbsi* is sister to* P. welaka*, and* P. harperi* is the basal-most sister species. In the sister* P. signipinnis* clade,* Pteronotropis signipinnis* is resolved as the basal sister group to remaining species.* Pteronotropis euryzonus* is likewise resolved as sister to a monophyletic* P. hypselopterus* complex with two sister clades ((*P. stonei* plus* P. metallicus*) sister to (*P. hypselopterus* (*P. merlini, P. grandipinnis*))). These two clades represent eastern and western groups, respectively, as defined by the Apalachicola River drainage (Figure 2). All ten major clades in the genus received high bootstrap and posterior probabilities (PP = 0.98–1.00), as similarly observed with analyses of nuclear genes by Mayden and Allen [8].

**Figure 3:**
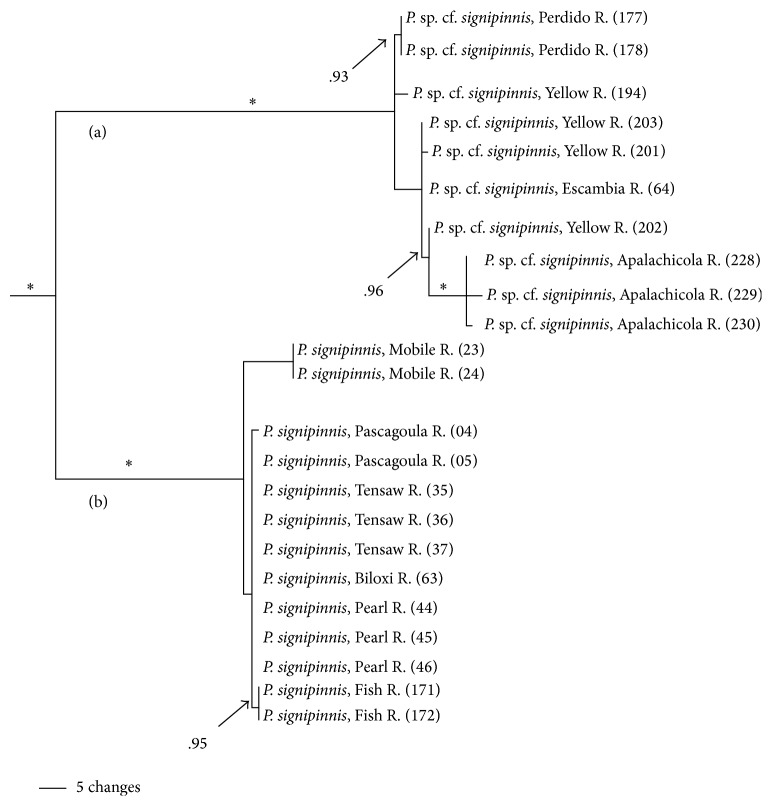
Relationships of populations in* Pteronotropis signipinnis* clade inferred from Bayesian analysis of ND2 sequences and summarized using a 50% majority rule consensus tree. *∗* = 100PP. (a)* Pteronotropis* sp. cf.* signipinnis* distributed east of the Mobile Basin. (b)* Pteronotropis signipinnis* distributed west of and from the Mobile Basin.

Considerable variation in ND2 sequence divergence exists across the range within the* P. signipinnis* clade with a mean sequence divergence of 10.7% (±2.6%). This is largely due to two subclades, each receiving maximum posterior probability support (PP = 1.0; Figure 3). The location of the Mobile Bay system geographically defined the boundaries of these two clades. Individuals from rivers west of and part of the Mobile Bay were recovered in one clade, herein referred to as the western* P. signipinnis* clade, whereas individuals from rivers east of the Mobile Bay formed the eastern* P. signipinnis* clade.

**Figure 4:**
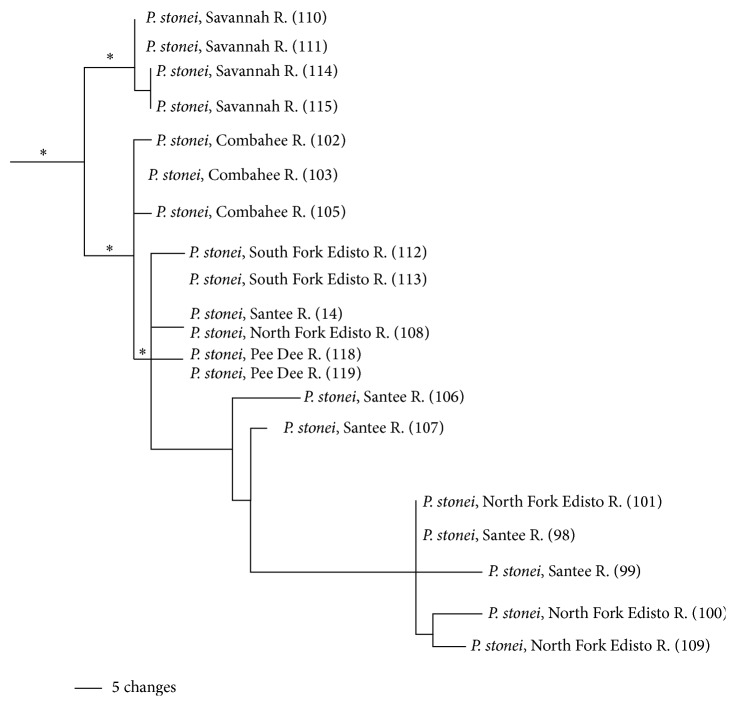
Relationships of populations in the* Pteronotropis stonei* clade inferred from Bayesian analysis of ND2 sequences and summarized using a 50% majority rule consensus tree. *∗* = 100PP.

No structure was observed within the* P. euryzonus* clade, likely due to its highly restricted range and the long-term impacts on the habitats of this species and its shrinking range [21]. Almost all individuals possessed the same haplotype for ND2; sequence was 0.0%. The* P. stonei* clade is distributed from the Savannah River in the south to the Pee Dee River in the north (Figures 1 and 4). Populations from the Savannah River formed a clade sister to other populations (PP = 1.0; Figure 4). The Combahee River populations formed the sister group to a clade including individuals from the North and South Forks of the Edisto, Santee, and Pee Dee rivers; populations from these river systems had little genetic structure. The overall within sequence divergence for the* P. stonei* clade was 3.3% (±1.0%).

**Figure 5:**
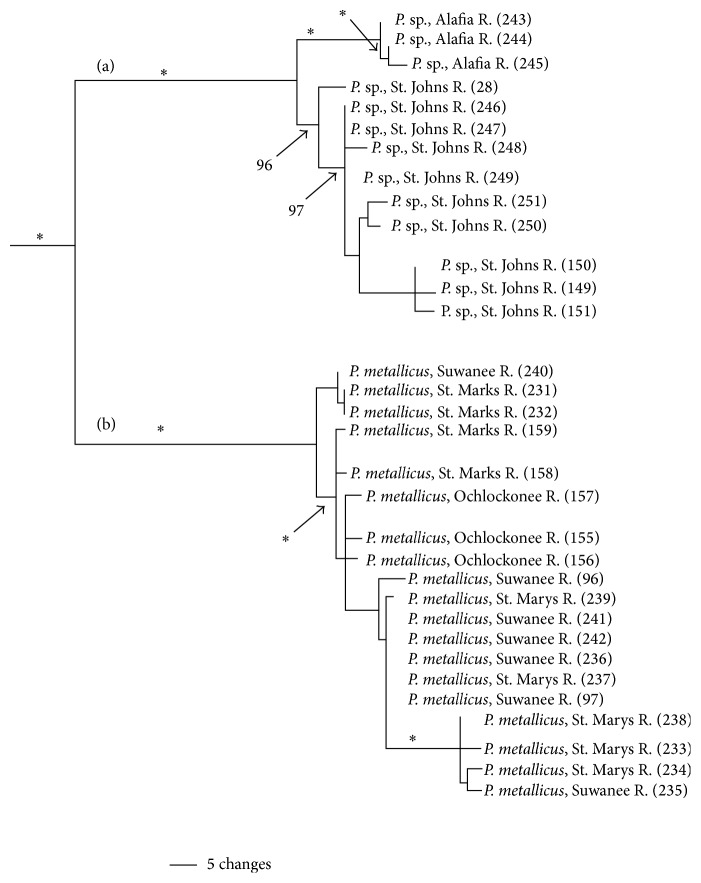
Relationships of populations in the* Pteronotropis metallicus* clade plus *P*. sp. cf.* metallicus* (a) inferred from Bayesian analysis of ND2 sequences and summarized using a 50% majority rule consensus tree. *∗* = 100PP.

The* P. metallicus* clade includes two major subclades (PP = 1.0; Figure 5). One clade includes only an undescribed species (*P.* sp. cf.* metallicus*) from the Alafia and St. Johns rivers. The second clade includes only* P. metallicus*, and both clades received strong support (PP = 0.1). Additional, strongly supported genetic structuring exists within both* P.* sp. cf.* metallicus* and* P. metallicus*. Structure within* P.* sp. cf.* metallicus* was strongly supported divergences between and within the Alafia and St. John's rivers (PP = 0.96–1.0); some drainage structure occurred in* P. metallicus* but not along independent drainages.* Pteronotropis metallicus* populations from the Ochlocknee, St. Marks, Suwannee, and St. Marys rivers had a haplotype diversity of 0.989 and a nucleotide diversity of 0.059 (Table 2). The St. Johns subclade includes populations from the St. Johns and Alafia Rivers and has a haplotype diversity of 0.987 and a nucleotide diversity of 0.010. The overall within group divergence for the* P. metallicus* clade was substantial (7.7% (±2.0%)), largely due to genetic differences between* P.* sp. cf.* metallicus* and* P. metallicus*.

**Figure 6:**
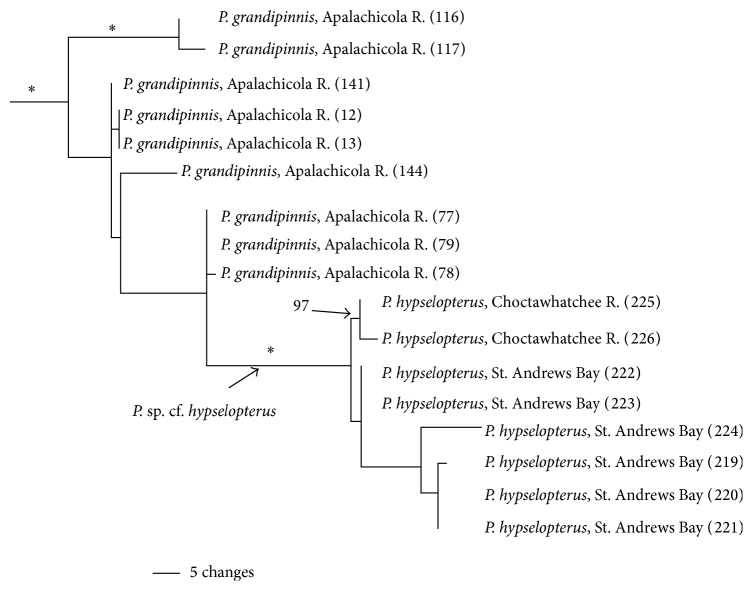
Relationships of populations in the* Pteronotropis grandipinnis* clade plus *P*. sp. cf.* hypselopterus* inferred from Bayesian analysis of ND2 sequences and summarized using a 50% majority rule consensus tree. *∗* = 100PP.

Given the low support for the relationship between* P. grandipinnis* to* P. merlini* and* P. hypselopterus* these three species clades should be considered an unresolved trichotomy (Figure 2). Support for the monophyly of* P. merlini* and* P. hypselopterus* and the* P. grandipinnis* clade was strong (PP = 1.0 for each). However, as currently outlined in the evolution of haplotypes* P. grandipinnis* is paraphyletic with respect to populations of* P. hypselopterus* from the Choctawhatchee and St. Andrews bays (PP = 1.0) (Figure 6). The overall within group variation for the* P. grandipinnis* clade was low (0.6%, ±0.4%).

**Figure 7:**
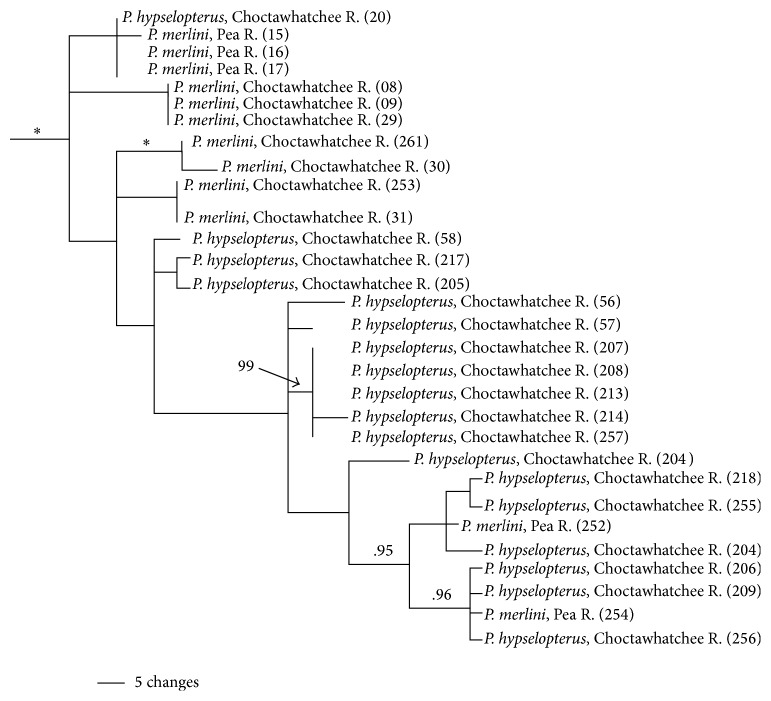
Relationships of populations in the* Pteronotropis merlini* clade plus* P.* sp. cf.* hypselopterus* inferred from Bayesian analysis of ND2 sequences and summarized using a 50% majority rule consensus tree. *∗* = 100PP.

**Figure 8:**
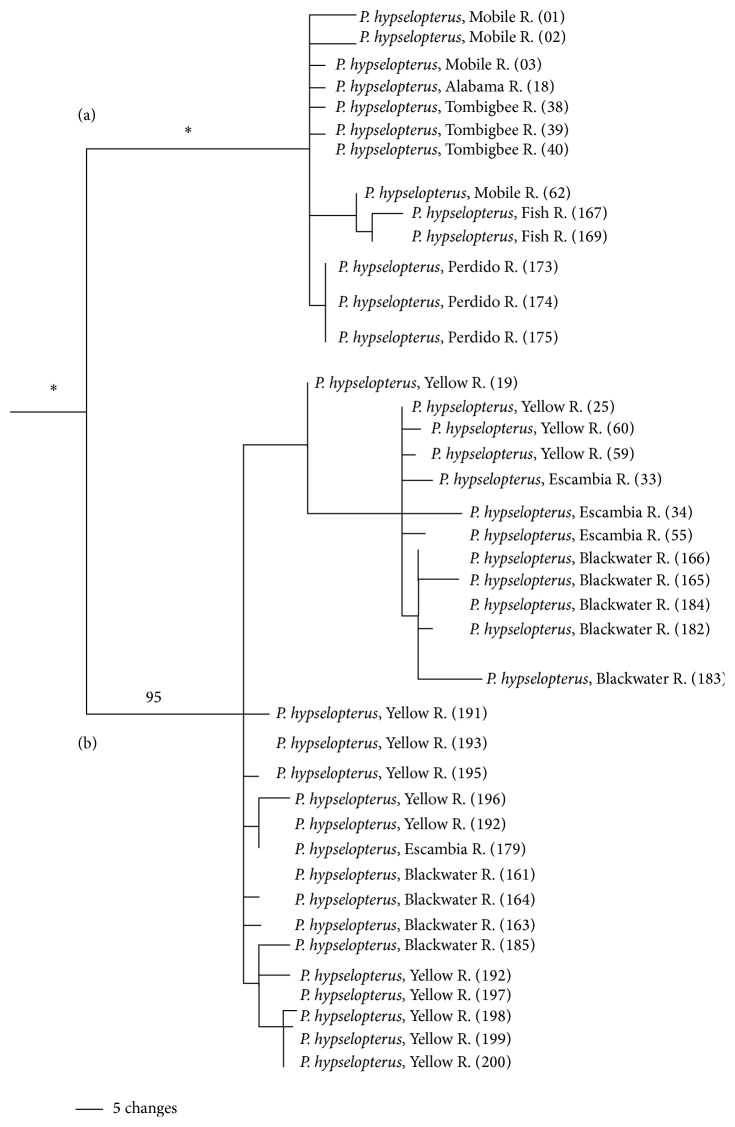
Bayesian 50% majority rule consensus tree for the relationships of populations in the* Pteronotropis hypselopterus* clade inferred from Bayesian analysis of ND2 sequences and summarized using a 50% majority rule consensus tree. *∗* = 100PP. (a) Clade of populations from rivers draining east and west of and from the Mobile Basin. (b) Clade of populations from rivers draining east of the Mobile Basin.

The* P. merlini* clade is strongly supported (PP = 1.0); however, similar to the* P. grandipinnis* clade,* P. merlini*, as currently outlined, is not monophyletic (Figure 7). Individuals of* P. hypselopterus* from the Choctawhatchee River drainage were resolved within the* P. merlini* clade. Resolution within this clade is limited and the within clade variation is low (1.6%, ±0.7%). There are, however, several well-supported subclades within this clade, one consisting of individuals of* P. merlini* from the Choctawhatchee (PP = 1.0), one* P. hypselopterus* from the Choctawhatchee River (PP = 0.99), and individuals of* P. hypselopterus* and* P. merlini*, also from within the Choctawhatchee (PP = 0.95). The* P. hypselopterus* clade is strongly supported (PP = 1.0) and includes two reciprocally monophyletic groups centered at the Mobile Bay, much like the* signipinnis* clade (Figure 8). Individuals from the Mobile Bay and associated rivers, the Alabama, Tombigbee, Fish, and Perdido rivers form one subclade (PP = 1.0) (herein referred to as the western* hypselopterus* clade). The other clade was also strongly supported and included individuals from the Yellow, Escambia, and Blackwater rivers (PP = 0.95) (herein referred to as the eastern* hypselopterus* clade). There was 4.8% (±1.2%) mean sequence divergence within the* hypselopterus* clade.

**Figure 9:**
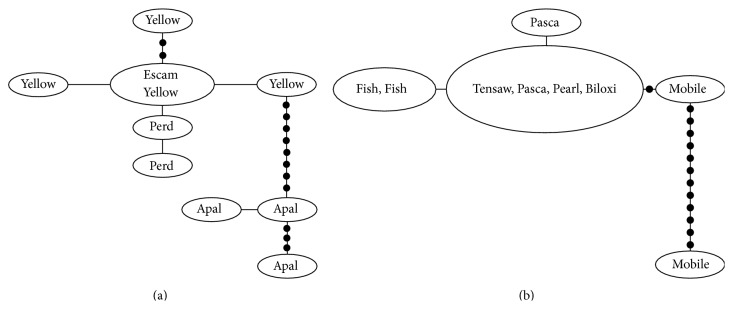
Haplotype network for* Pteronotropis signipinnis* clade. (a)* Pteronotropis* sp. cf.* signipinnis.* (b)* Pteronotropis signipinnis.* Apal = Apalachicola River system, Biloxi = Biloxi River system, Escam = Escambia River system, Fish = Fish River system, Pasc = Pascagoula River system, Pearl = Pearl River system, Perd = Perdido River system, Mobile = Mobile River system, Tensaw = Tensaw River system, and Yellow = Yellow River system.

**Figure 10:**
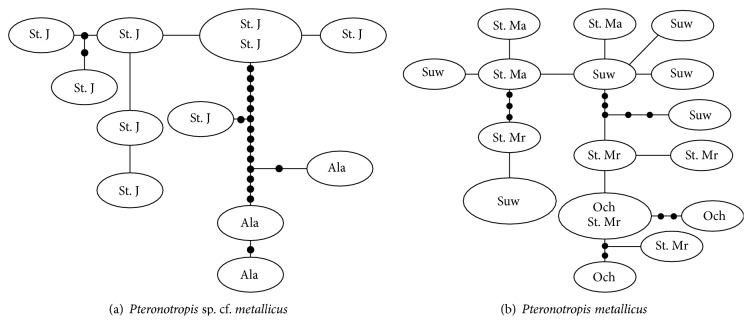
Haplotype networks for (a)* P.* sp. cf.* metallicus* and (b)* P. metallicus*. Ala = Alafia River system, Och = Ochlockonee, St. J = St. John River system, St. Ma = St. Marys River system, and Suw = Suwanee River system.

**Figure 11:**
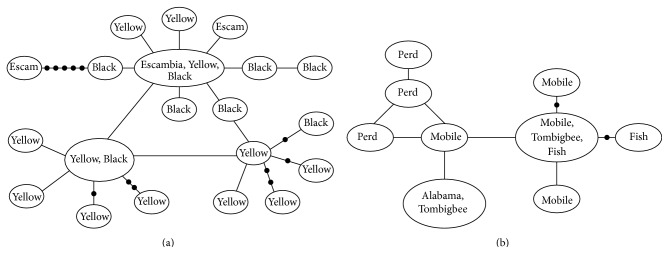
Haplotype networks for* Pteronotropis hypselopterus*. (a) Clade of populations from rivers draining east of Mobile Basin. (b) Clade of populations from rivers of the Mobile Basin and adjacent and more western river systems.

**Figure 12:**
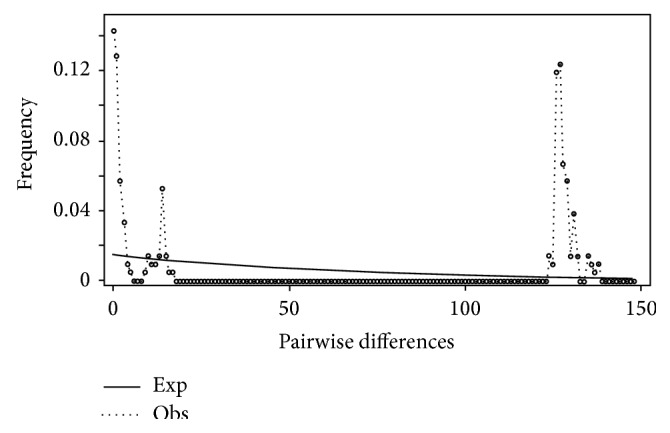
Mismatch distribution of* Pteronotropis signipinnis* clade.

**Figure 13:**
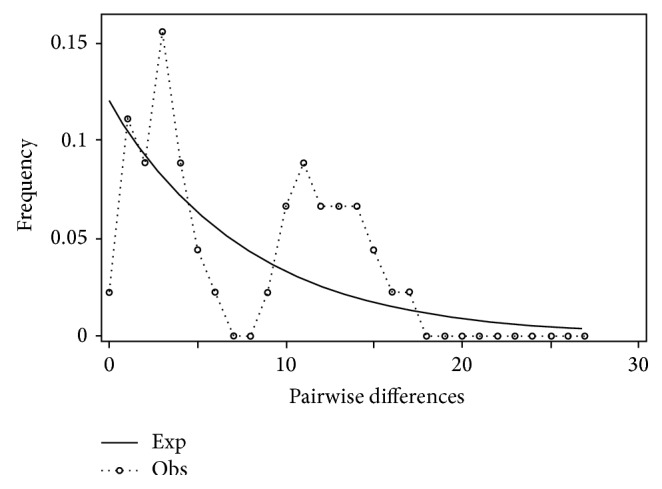
Mismatch distribution of eastern* Pteronotropis signipinnis* clade.

**Figure 14:**
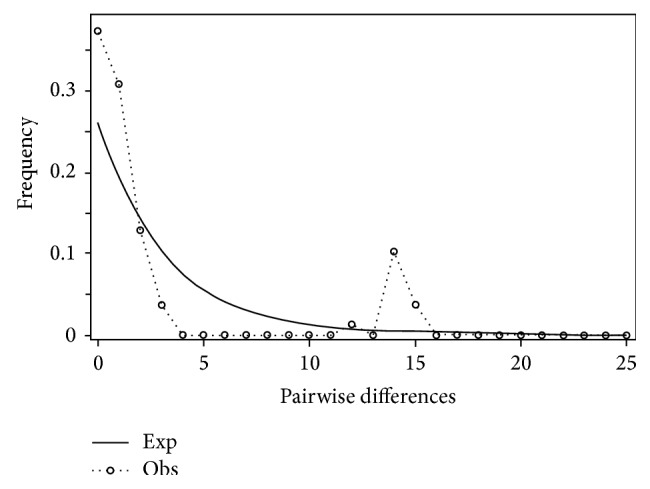
Mismatch distribution of western* Pteronotropis signipinnis* clade.

**Figure 15:**
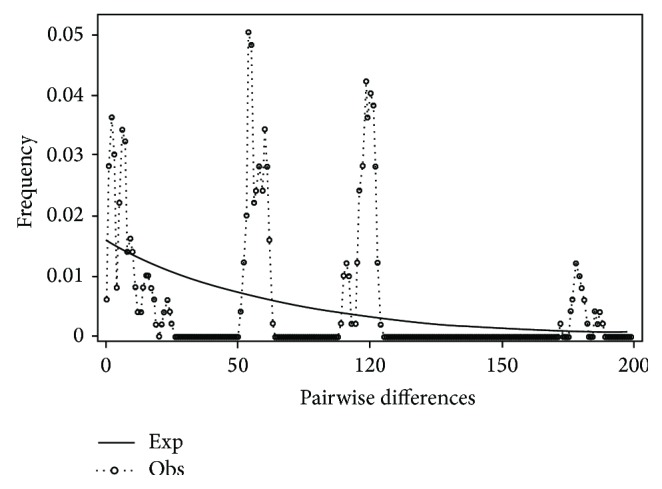
Mismatch distribution of* Pteronotropis metallicus* clade.

**Figure 16:**
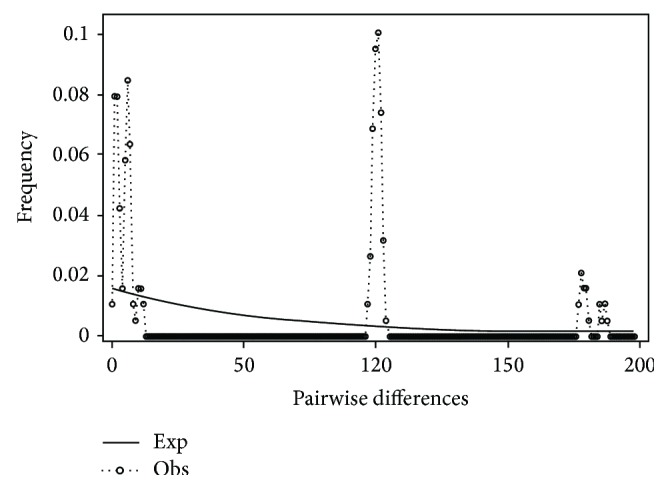
Mismatch distribution of the* Pteronotropis metallicus* clade excluding individuals from the St. Johns and Alafia Rivers.

The lineages of* P. signipinnis*,* P. euryzonus*,* P. metallicus*,* P. stonei*,* P. grandipinnis*,* P. merlini*, and* P. hypselopterus* are examined in more detail in TCS analysis (Figures 9–16). The algorithm was unable to connect the eastern and western* signipinnis* clades, the* P. metallicus* subclades from the Ochlocknee and St. Johns, or the eastern and western* hypselopterus* clades at a 95% connection limit (21 steps; Figures 9–11). Within the eastern* signipinnis* clade nine haplotypes were recovered as well as strong geographic partitioning within this clade. Individuals from the Escambia, Yellow, and Perdido rivers clustered together and were nine mutational events apart from the three haplotypes found in individuals from the Apalachicola River. The western* signipinnis* clade included more individuals but included only five haplotypes. Many individuals from the Tensaw, Pascagoula, Pearl, and Biloxi rivers share a common haplotype with one individual from the Mobile Bay having a haplotype thirteen mutational steps from all others (Figure 9). For the Ochlocknee River* P. metallicus* clade, 16 haplotypes were recovered from 30 individuals. In this clade there was little structure with most individuals having similar haplotypes (Figure 10(b)). This is in contrast to the St. Johns River clade that displayed significant structure (Figure 10(a)). Individuals from the St. Johns River clustered together, having 13–15 mutational steps from the three haplotypes in the Alafia River. Furthermore, within the St. Johns River clade, 11 haplotypes were recovered from 12 individuals. Both the eastern and western* P. hypselopterus* clades displayed limited geographic partitioning, with 20 recovered haplotypes from 39 individuals from the eastern* hypselopterus* clade and nine haplotypes from 13 individuals within the western* hypselopterus* clade (Figure 11).

**Figure 17:**
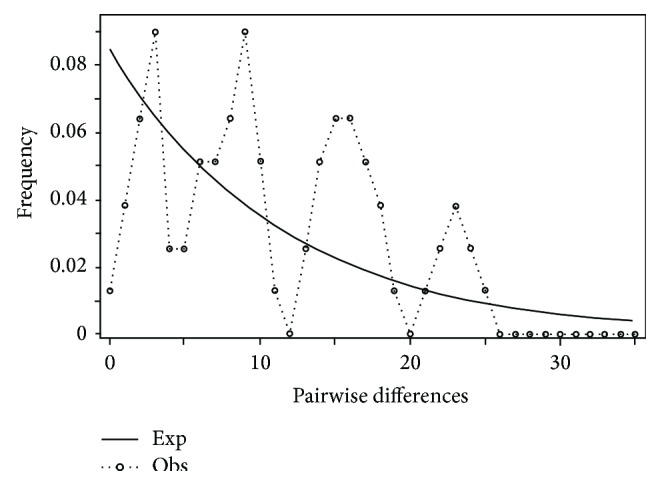
Mismatch distribution of the* P.* sp. cf.* metallicus* clade.

**Figure 18:**
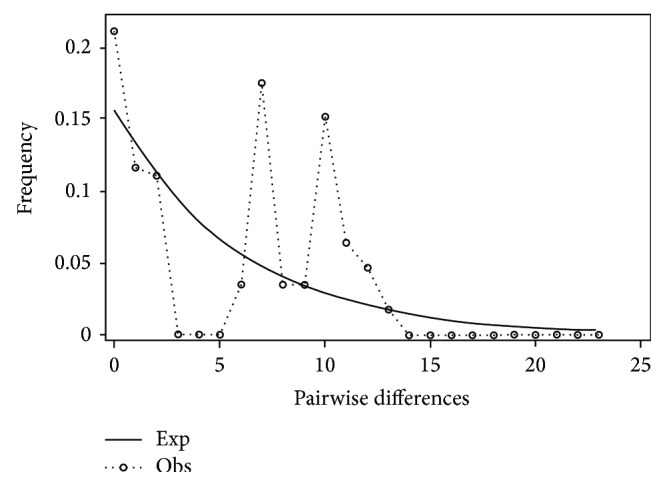
Mismatch distribution of the* Pteronotropis stonei* clade.

**Figure 19:**
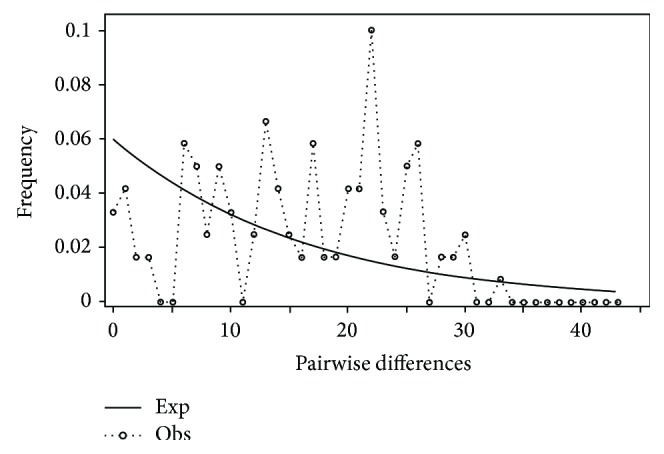
Mismatch distribution of the* Pteronotropis grandipinnis* clade.

**Figure 20:**
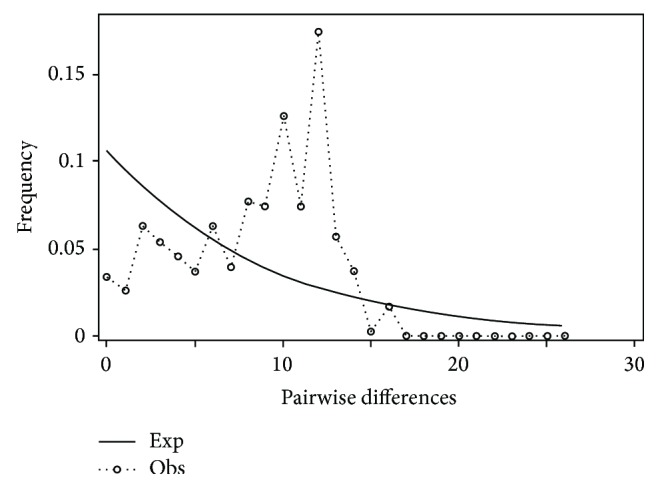
Mismatch distribution of the* Pteronotropis merlini* clade.

**Figure 21:**
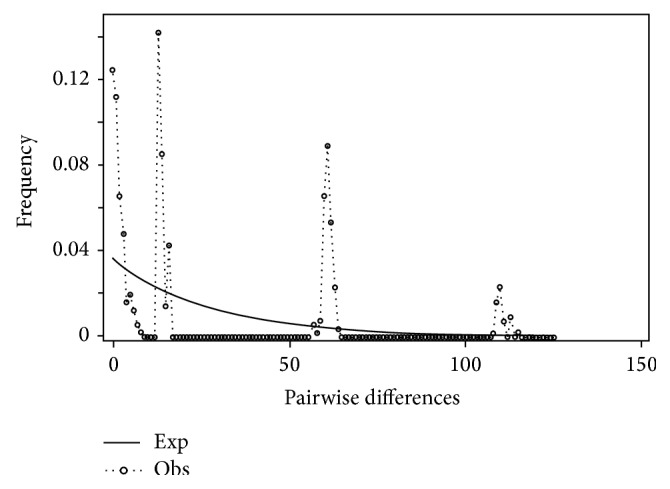
Mismatch distribution of the* Pteronotropis hypselopterus* clade.

Mismatch distribution plots for nearly all recovered clades had multimodal distributions, clearly fitting the model of nonexpanding populations or having populations at equilibrium (Figures 12 and 15–21). These results are supported by the lack of significance found in the Tajima's *D* test (Table 2). The one exception was the* signipinnis* clade (eastern and western clades combined). This clade had a bimodal distribution, indicating a stable population ([13, 14], Raggedness index = 0.288); however, Tajima's *D* test statistic was significant for a rapid population expansion (Table 2). One possible explanation for the conflicting results is that the* signipinnis* clade consisted of two distinct populations with likely independent demographic histories. To account for this, the clades that showed well-supported subclades (*P. signipinnis*,* P. metallicus,* and* P. hypselopterus*) were analyzed as partitioned data sets. Within this framework the western* signipinnis* clade displayed a unimodal distribution, corroborating population expansion (Figure 13), a hypothesis also supported by significance for Tajima's *D* test (Table 2).

## 5. Discussion

### 5.1. Overall Phylogeographic Structure

Findings in this study were consistent with previous analyses rejecting the monophyly of* Pteronotropis* if* P. harperi* continues to be placed in* Notropis* [8, 25–28]. In the BA tree, two major clades were resolved for the genus. The first included* P. hubbsi*,* P. welaka*, and* P. harperi*, the* Pteronotropis harperi* clade. The second contained* P. signipinnis* as the basal sister group* P. euryzonus* and members of the* P. hypselopterus* complex and* P. hypselopterus* (Figure 2), the* P. hypselopterus* clade. These relationships were well supported except for Node A (Figures 2 and 3); these three clades above this node, with this dataset, should be considered a trichotomy. These sister group relationships were also recovered using two nuclear gene loci by the authors [8]. Given the understanding of species relationships revealed by the BA tree and two nuclear gene loci, the discussion below focuses on phylogeographic patterns within* P. signipinnis*,* P. euryzonus*, and the* P. hypselopterus* complex.

One of the most important discoveries to come out of vicariance biogeography was the realization that a broad range of taxa, within a defined geographic area, will show similar distribution patterns, and likely similar sister group relationships, due to shared historical geological events isolating common ancestors across clades in the region. These geological events that impede gene flow and lead to strong intraspecific breaks among populations eventually lead to lineage splitting or cladogenesis. These replicated patterns are useful in comparative and evolutionary biology because they provide a null hypothesis or strong* a priori* predictions for unsampled taxa with similar distributions in a given area. Further, they can aid in conservation and management by delineating evolutionary significant units (ESUs) or cryptic species that might otherwise go unnoticed. The Bayesian tree structure (Figure 2) supports many phylogeographic patterns seen by other authors in the Coastal Plain of the southeastern United States, as well as some likely undescribed species.

Suttkus and Mettee [7] hypothesized that* P. signipinnis* had its origin in the upper Tombigbee system. The data presented here cannot refute this hypothesis.* Pteronotropis signipinnis* is resolved as sister to* P. euryzonus* and the* P. hypselopterus* complex (Figure 2) with high (17.4–19.5% uncorrected; 18.0–22.6% corrected) sequence divergence between it and the other species. Assuming the rate of evolution in the ND2 gene is similar between log perch darters (Percidae:* Percina*) and* Pteronotropis*, 2.0% per million years, as calculated by Near and Benard [43] and used in another study on minnows by Berendzen et al. [44], this would give a minimum divergence time for* P. signipinnis* of 22.6 million years before present (MYBP), roughly in the early Miocene. After an abrupt lowering of sea levels during the late Oligocene, sea levels rose rapidly (80–100 meters above present level) in the early Miocene and continued at this level, with some minor drops, for much of the Miocene [4]. Two of the rivers along the Gulf Slope that would have remained distinct and have separate outflows to the Gulf of Mexico at that time were the Tombigbee and Chattahoochee rivers. One scenario is that a contiguous ancestral population was split into two populations by the rise in sea level, leaving two disjunct populations, one in the then Tombigbee River and the other in the then Chattahoochee River. Presumably the ancestral populations in the Tombigbee River, through lineage splitting, eventually gave rise to* P. signipinnis* while the populations in the Chattahoochee River were ancestral to* P. euryzonus* plus the* hypselopterus* complex [7] and underwent subsequent isolation and divergence. Following another lowering of sea levels during this global cycle, dispersion of ancestral forms down these rivers that were now connected further out from the present coastline in the Gulf Slope would have provided for their movement to other river systems as habitat became available. This hypothesized historical biogeographic scenario involving both dispersal and vicariance is further supported by the fact that* P. euryzonus* is resolved, with high support, as the sister group to the diverse* P. hypselopterus* complex (Figure 2), and remains endemic to the middle and lower Chattahoochee River.

During the high sea stands of the Miocene, the Chattahoochee River is thought to have served as refugial habitat for populations of many freshwater fishes of the Gulf Slope resulting from inundation of waterways by the Gulf of Mexico and Atlantic Ocean [4]. Both the Chattahoochee and Flint rivers, as part of the same drainage system, flow in a southerly direction into Lake Seminole; the Apalachicola emerges from Lake Seminole and discharges into the Gulf of Mexico. This river system is known to have been and continues to be a major barrier to gene flow for populations of the same species with distributions to the east and west of the Apalachicola River. The distinctiveness of the diversity and divergence east and west of this drainage is renowned, so much so that Avise [1] devoted a large portion of his chapter on geological concordance to describing its history. Some of the organisms that show this break include reptiles [45], amphibians [20–46], fishes [3, 4, 47–50], macroinvertebrates [41], spiders [51], and trees [52]. The data presented here further corroborate these multilineage findings.

Within* Pteronotropis*, a clade containing* P. stonei*,* P. metallicus*, and* P. *sp. cf.* metallicus* is resolved as sister to clade wherein undescribed species may exist as separate lineages and where* P. merlini* +* P. hypselopterus* are sister to* P. grandipinnis.* All populations of* P. stonei* and* P. metallicus* occur to the east of the Apalachicola,* P. grandipinnis* occurs in the Apalachicola, and* P. merlini* and* P. hypselopterus* occur west of this drainage. The nodal support for the sister group relationship in this east-west split between* P. grandipinnis* and the* P. merlini* +* P. hypselopterus* clade is not strong (PP = 0.53; Node A, Figure 2); however, the nodes supporting the sister group relationship between P. grandipinnis plus* P.* sp. cf.* hypselopterus* as well as that for* P. merlini* +* P.* sp. cf.* hypselopterus* and* P. hypselopterus* are strongly supported by nuclear genes (PP = 1.0) (Figure 2 [8]).

Another evidence in support of* P. grandipinnis* being closely allied with the western group (*P. merlini* +* P.* sp. cf.* hypselopterus* and* P. hypselopterus*) comes from morphological data. Suttkus et al. [24] diagnosed* P. stonei* (eastern group) as having a dark lateral band continuous to the base of the caudal fin without any intensification at the base of the caudal fin, a ventral margin of the lateral band with a clearly defined border, and nuptial males of* P. stonei* lacking enlarged dorsal and anal fins. This is in contrast to* P. grandipinnis* and others of the western group which show an intensification in pigment in their lateral band at the caudal fin, a diffuse ventral margin of the lateral band, and slightly to greatly elevated dorsal and anal fins in nuptial males (especially in* P. grandipinnis*). As outlined by Avise [1] agreement between gene trees and other biogeographic data (aspect IV, genealogical concordance) provides assurance that gene trees can register these phylogeographic breaks.

### 5.2. *Pteronotropis signipinnis* Clade

All analyses of this group indicate that cryptic species diversity exists within* P. signipinnis*. Two reciprocally monophyletic clades are recovered (each with 100% bootstrap support and PP = 1.0) (Figures 2 and 3) within this species. Further, there is highly significant sequence divergence (10.7%), haplotype diversity, and nucleotide diversity (Table 2) within this clade.* Pteronotropis* sp. cf.* signipinnis* from the eastern* signipinnis* clade is a distinct taxon under both the Evolutionary Species Concept as the theoretical concept [53] and the Phylogenetic Species Criterion as the operational method for discovering evolutionary species as lineages [54–60]. The divergence between* P. signipinnis* and* P.* sp. cf.* signipinnis* is consistent with other taxa inhabiting the same area [3, 23, 61]. Bailey and Suttkus [62], in their description of* P. signipinnis*, observed differences in meristics between populations on either side of the Mobile Bay.

Higher levels of genetic variation in both haplotype and nucleotide diversity were also observed in* P.* sp. cf.* signipinnis* relative to* P. signipinnis* (Table 2). Most individuals in the* P. signipinnis* clade share a common haplotype, but those in* P*. sp. cf.* signipinnis* possess many unique haplotypes (Figure 9). At least three possible historical events may account for the lack of genetic variation within* P. signipinnis*. It could be the result of a genetic bottleneck or recent populations in and to the west of the Mobile Bay experienced a rapid population decline leading to the rapid fixation of only a few haplotypes. Given the current data these two alternative hypotheses cannot be differentiated or tested. Possibly a more plausible scenario, based upon the differences between populations east and west of the Mobile Bay, is that of founder affect. As shown in the mismatch distribution plot (Figure 14),* P. signipinnis* has undergone a recent range expansion, supported by a significant value for Tajima's* D* (Table 2). Genetic diversity in comparisons of individuals from the Mobile R. and Bay is notable with thirteen mutational steps (Figure 9). In this example, one possibility is that a haplotype yet to be found in the Mobile rivers was transported via dispersal, through stream captures or some other means, to the Escatawapa or Pascagoula rivers as habitat became available during a post-Pleistocene lowering of sea levels. This explanation is not without merit as many studies have demonstrated that mitochondrial genes are good markers for detecting range expansion of species or populations [15, 41, 63–67]. One interesting aspect of the data and analyses presented herein, however, is that no other clade within* Pteronotropis* shows evidence of a recent population expansion (Figures 12-13, 15–21). This seems to indicate that the western dispersal of* P. signipinnis* was much more recent than the possible migration of other members in the genus. Several studies of North American fish species have detected rapid range expansion in the examination of taxa from rivers of the Central Highlands, all presumably following glacial fronts and colonizing rivers in glaciated areas of the Central Lowlands as glaciers moved northward [15, 42, 63–69]. Conditions in Gulf Slope rivers, however, were quite different than those that once impacted the cold-water rivers of the Central Highlands. Swift et al. [4] predicted that if taxa from rivers of the Gulf Slope dispersed it would have been at the beginning (not the end, as in Central Highland taxa) of the Pleistocene, a difference of about 1.81 million years. A very real possibility exists that the mitochondrial ND2 gene marker does not possess adequate variation in* Pteronotropis* to detect early population expansions. To address this question would require examination of microsatellites amongst many populations.

### 5.3. *Pteronotropis euryzonus* Clade

Having strong lineage support (BS = 100%, PP = 1.0)* Pteronotropis euryzonus* is resolved as the basal sister group to this* P. hypselopterus* clade (Figure 2), a relationship corroborated in other studies using either morphology or molecular data [70–72], but not in the nuclear gene phylogeny of Mayden and Allen [8] where* P. euryzonus* is resolved as the sister species to a clade consisting of* P. metallicus* +* P. stonei*. In the description of* P. euryzonus*, Suttkus [73] described two morphological races within this species, a northern race termed the Uchee race and a southern Chattahoochee race from the lower portions of that river. Data presented here can neither corroborate nor refute this hypothesis because samples used herein were from the upper portions of the Chattahoochee River drainage. Warren et al. [74] listed* P. euryzonus* as a species of special concern, due to its limited range, and the results of this study indicate little genetic variation within the sampled populations. All individuals of the three populations examined from Snake, Maringo, and Uchee creeks have the same haplotype, and a within species sequence divergence of 0%, a haplotype diversity of 0.464, and a nucleotide diversity of 0.00 (Tables 2 and 3). These results emphasize the recommendations of Boschung and Mayden [21] that periodic population monitoring of known localities of this species should be a priority.

### 5.4. *Pteronotropis stonei* Clade

This clade is found in streams draining the Atlantic Slope, including the Pee Dee, Santee, North and South Fork Edisto, Combahee, and Savannah rivers. Suttkus et al. [24] elevated* P. stonei* from synonomy of* P. hypselopterus* and hypothesized that the species was closely related to* P. metallicus* but provided no phylogenetic evidence. Molecular variation and analyses presented here corroborate this hypothesis (Figure 2), because* P. stonei* and* P. metallicus* are reciprocally monophyletic and sister species herein and with nuclear genes [8]. The* P. stonei* clade and some subclades of the gene tree were highly supported (BS = 100%, PP = 1.0; Figure 4).

Mitochondrial variation supports the hypothesis of range expansion and speciation within this complex originating and centered about the Chattahoochee and Apalachicola river systems. Using the logic of molecular divergence employed above, sequence divergence between* P. euryzonus* and* P. stonei* (10.1%, Table 3) would support a divergence time of about 10.1 MYA. Assuming a 2% molecular clock, this places this speciation event at mid-Miocene during a period of rising sea levels [4], potentially isolating populations in headwater streams and opportunity for divergence. The close level of divergence of* P. stonei* and* P. metallicus* (9.6%) from* P. euryzonus* (10.1%) would suggest that the ancestral populations had already spread eastward into the Ochlocknee and across the Atlantic coastal streams, thus creating multiple, refugial populations in the ancestor. The lowering of sea level during the late Miocene altered drainage patterns in their lower reaches and resulted in the connection of multiple formerly isolated basins on the continental shelf. This expanded the coastal plain, creating habitats identical to those currently inhabited by species of* Pteronotropis* and is herein hypothesized to have provided for a northern expansion of populations. This is consistent with mismatch analysis, and such an expansion may have occurred via the Tifton uplift in southern Georgia or the Ocala uplift in northern Florida, both thought to have been distinct since the Eocene [22]. Opportunities for other taxa to move into eastern streams may have existed at about this time. For example, populations of* Pteronotropis welaka*,* Lepisosteus oculatus*, and* Opsopoeodus emiliae* occur on both sides of the Apalachicola River, but their ancestral populations are thought to have existed west of this drainage [7]. For instance,* P. welaka* is the sister species of* P. hubbsi,* an endemic known in the Mississippi River valley from southern Illinois (now extirpated) and in the Little and Ouachita rivers in southern Arkansas and northern Louisiana, west of the Apalachicola. Studies of other freshwater fish species (Near et al. [75]; Roe et al. [61]) from these same drainages of the Gulf Slope have estimated similar speciation dates.

### 5.5. *Pteronotropis metallicus* Clade

The* P. metallicus* clade (Figure 5) contains two strongly supported major reciprocally monophyletic subclades (100% bootstrap, PP = 1.0; Figure 5). Overall within species sequence divergence in* P. metallicus* is 7.7% and is largely due to the presence of the two major subclades (Figure 5(b)). With the high support for these subclades and the large amount of sequence divergence between them, invoking the Phylogenetic Species criterion under the Evolutionary Species Concept as an overriding concept [76], we recognize this lineage as an undescribed species (Figure 5(a), St. Johns subclade). This divergence was also discussed by Suttkus [73] but the lineage was not officially named. The species can also be diagnosed using morphological traits [73] and was originally thought to occur in the Withlacoochee, Hillsborough, St. Marys, and St. Johns rivers. Subsequently, all populations in St. Marys River were referred to* P. metallicus* [24]. Suttkus [73] noted distinct populations of* P. hypselopterus* from the Alafia River (see Figure 5(a)) and recommended this as a distinct subspecies. The hypothesized diversity identified by Suttkus [73], herein recognized as species, is supported by current molecular data and analyses. Within the St. Johns subclade (Figure 5(a)) least two distinct genetic and morphologically diagnosable lineages exist, one in the Alafia River system (100% BS, PP = 1.0) and one in the St. Johns River system (PP = 0.96; Figures 5 and 10). TCS analysis (Figure 10) identifies populations in the Alafia River system as being fifteen mutational steps away from populations from the St. Johns River system. Separation of populations from the Alafia River from populations in the St. Johns River is predicted to have been fairly recent as TCS analysis can connect these two populations within a 95% connection limit.

### 5.6. *Pteronotropis grandipinnis*—“*P. hypselopterus*” Clade

*Pteronotropis grandipinnis* is sister to a clade inclusive of populations of* P. merlini* plus some populations of* P. hypselopterus* (Figure 2 node A and Figure 6). In this clade* P. hypselopterus* is not resolved as monophyletic as individuals of* P. hypselopterus* from St. Andrews Bay and Choctawhatchee Bay drainages were resolved as a subclade (100% bootstrap, PP = 1.0) within* Pteronotropis grandipinnis*. Constraining the gene tree of* P. hypselopterus* or* P. grandipinnis* as monophyletic groups, respectively, resulted in significantly worse ML tree scores than the best trees (*P* = 0.0002) using the Shimodaira and Hasegawa [77] test. This clade of specimens of “*P. hypselopterus*” from these drainages recovered within* P. grandipinnis* may be either an instance where the gene tree does not accurately reflect the species tree or clade or these “*P. hypselopterus*” represent an undescribed species (*sensu* the Evolutionary Species Concept [53–55, 57–59, 76]). With regard to the first possibility, two potential explanations may account for the pattern seen in this clade, introgression between* P. hypselopterus* and* P. grandipinnis*, or incomplete lineage sorting of haplotypes within an ancestral species having an ancestral polymorphism [78]. Given the current data, it is impossible to distinguish between these alternatives. However, given that the* P. hypselopterus* clade has high support it likely indicates that this group of “*P. *sp. cf.* hypselopterus*” represents a different undescribed species. Currently the headwater tributaries of the St. Johns Bay river system and the Apalachicola River system are very close in air miles. For instance, the authors collected many individuals of* P. hypselopterus* at Bear Creek (tributary to St. Johns Bay drainage), which has its headwaters less than two air miles from the headwaters of Juniper Creek, a tributary of the Chipola-Apalachicola Rivers. Because both of these creeks flow through lowland cypress swamps, it is possible that these systems were connected one or more times in the evolution of this lineage (Figure 6). Given that the gene tree for the* P*. sp. cf.* hypselopterus* is highly divergent and monophyletic and the more basal specimens from the Apalachicola River have limited resolution, it is likely that this gene provides only some resolution and that other genetic markers are needed. It is possible that the populations from the Apalachicola River,* P. grandipinnis*, is a natural grouping with a monophyletic gene tree. Further analyses using alternative genes and finer scale markers such as microsatellites are needed for further resolution to aid in distinguishing between alternative explanations.

### 5.7. *Pteronotropis merlini*—“*P. hypselopterus*” Clade

As with the evolutionary relationships among populations of* P. grandipinnis,* the gene tree for* P. merlini* did not resolve all specimens of this species as closest relatives. Rather, some haplotypes of specimens of* P. merlini* were recovered as being more closely related to specimens of* P. hypselopterus* from the Choctawhatchee River drainage than to other* P. merlini* from the same drainage (Figure 7). While these relationships were resolved, there are no supporting values for basal nodes and some nodes between* P. merlini* and* P. hypselopterus* from the Choctawhatchee River; however, some nodes supporting monophyly of the gene tree for many individuals from the Choctawhatchee River are strongly supported. Constraining gene trees for* P. hypselopterus* or* P. merlini* as monophyletic resulted in significantly worse ML tree scores than the best trees (*P* = 0.0002) using the Shimodaira and Hasegawa [77] test. Some may question the validity of* P. merlini* due to its lack of genetic distinctiveness and its possible lack of evolutionarily independence from* P. hypselopterus* in the Choctawhatchee River Basin for the mitochondrial gene ND2. In this situation, unlike that in* P. grandipinnis,* the haplotypes of these* P. hypselopterus* are not clustered into a highly supported clade but are dispersed (Figure 7). Analyses do strongly support the monophyly of the gene tree uniting* P. merlini* and* P. hypselopterus* from the Choctawhatchee River, clearly indicating that these* P. hypselopterus* are not closely related to the others species occurring in different clades. Testing the relatedness of these populations and the monophyly of the gene tree for* P. merlini* requires additional genes and would benefit from microsatellite analyses. It is possible that the gene tree resolved in this pattern can be explained without invoking an active process being involved within the Choctawhatchee River drainages following the most recent common ancestor of the* P. grandipinnis* plus “*P. hypselopterus*” clade. Other than the simple lack of resolution using ND2 sequence variation, active process-free explanations following the divergence could be result of either incomplete lineage sorting in a shared ancestral population to both* P. merlini* and “*P. hypselopterus*” from the Choctawhatchee River or specimens/populations of* P. hypselopterus* from the geographic area in question retaining haplotype polymorphisms in their most recent common ancestor.

*Pteronotropis merlini* inhabits more upland habitats in this drainage, and* P. hypselopterus* occurs in more lowland habitats below the confluence with the Pea River [7], ecological and behavioral predispositions that may limit their geographic overlap. Further, morphological characteristics exist to distinguish the two species, including differences in body depth (*P. merlini* has a deeper body), orange coloration in the caudal fin of* P. merlini* versus olive-yellow coloration in* P. hypselopterus*, and the chevron-lunate shaped blotch on the caudal fin separated from the dark lateral band in* P. merlini* that is lacking in* P. hypselopterus*. These features argue for the independence of the two groups from this region and do serve as counter evidence for any ongoing gene flow, although morphology can be a poor surrogate for evidence of gene exchange [79–82]. Upon close examination no morphological intermediates have yet to be found between these two species* P. merlini* and* P. *sp. cf.* hypselopterus*. Further, no specimens morphologically identifiable as* P. merlini* have ever been taken downstream of the confluence of the Pea and Choctawhatchee rivers nor have any specimens morphologically identifiable as* P. hypselopterus* been taken upstream of this confluence. Additional morphological and more fine-scaled molecular data are needed in appropriate analyses to examine the possibility that populations of* P. *sp. cf.* hypselopterus* from the Choctawhatchee River drainage do not represent a distinct lineage. As multiple new species have been described or detected across the widespread distribution of the formerly recognized* P. hypselopterus*, additional study using different markers of varying degree of potential anagenesis and detailed morphological study remain as possible tests to the hypothesis of the two lineages in the Choctawhatchee River.

### 5.8. *Pteronotropis hypselopterus* Clade

In no gene tree of ND2 was a clade composed exclusively of currently recognized populations of* P. hypselopterus* resolved as monophyletic (Figures 2, 6, and 7). Some populations were found to be more closely related to* P. grandipinnis* (Figure 6) or* P. merlini* (Figure 7). For most specimens of* P. hypselopterus* gene tree analysis identified a strongly supported (100%) monophyletic group with two well-supported and geographically defined independent groups with their distributions being east and west of the Mobile Bay (Figures 8 and 11), much like the pattern and relationships observed in* P. signipinnis*.

Other taxa have their distributional limits delineated east and west of the Mobile Bay [3, 25, 62]. The clear distinctiveness of taxa on either side of the Mobile Bay has been explained by elevated sea levels that isolated populations of species in the headwaters of rivers east and west of the Mobile Bay. After the subsequent lowering of sea levels during the mid- to late-Miocene, drainage flow of the Alabama-Tombigbee Rivers turned southward (from west or south-westward) further isolating populations on either side of the bay [3, 59]. However, these historical events do not fit the time signature seen in* P. hypselopterus*, if a constant molecular time divergence assumption hypothesis is valid. Assuming a molecular clock of 2% sequence divergence per million years [4, 44] the 4.8% within sequence divergence observed in* P. hypselopterus* would correspond to ~4.8 MYBP or roughly in the mid-Pliocene.

Haplotype diversity and structure differ between the eastern and western clades of* P. hypselopterus*. The eastern* P. hypselopterus* clade has high haplotype diversity (Table 2) but shows little genetic structuring relative to the hierarchical structuring of drainages (Figure 11). Many haplotypes are shared between the Escambia, Yellow, and Blackwater rivers. These river systems have few endemics and other freshwater fishes show similar distributional patterns in these systems [3]. The western clade of* P. hypselopterus* possesses two main haplotype clusters (Figure 11). One cluster includes haplotypes from the Perdido River group with those of the Mobile, Alabama, and Tombigbee rivers; the other cluster includes haplotypes from populations in the Fish and parts of the Mobile and Tombigbee rivers. Although two clusters of haplotypes occur in the western* P. hypselopterus* clade, they differ only by a single mutation, as indicated by low haplotype and nucleotide diversity (Table 2). Due to the low degree of within sequence divergence in the* P. hypselopterus* clade (compared with similar taxa in the region) and the apparent limited morphological distinctiveness between populations [7], we recommend no taxonomic changes. However, these populations warrant further study with additional more highly variable genetic markers and more detailed examination of both museum and live and breeding adults from all of the rivers to resolve potential lineage divergence or mixing within this clade. The limited divergence patterns observed in this clade and between the eastern and western clades may be due to recent divergences, a mismatch of appropriate genes and lack of detailed morphological studies of coloration of live and breeding adults, or simply a depressed rate of anagenesis in the* P. hypselopterus* lineage (excluding those populations that are more closely related to either* P. grandipinnis* or* P. merlini*).

## 6. Conclusions

Phylogenetic analysis of populations and species of* Pteronotropis* reveal multiple new hypotheses regarding the monophyly of genes, species diversity, potential undescribed species, and abiotic factors correlated with divergence events between and within species. These findings fully support those of earlier studies (Suttkus and Mettee [7], Suttkus et al. [24], Bailey and Suttkus [62], and Suttkus [73]) which were all based on morphological data.* Pteronotropis* is a monophyletic genus but only with the inclusion of* Pteronotropis harperi*, a species that has long had unresolved relationships. Findings and hypotheses herein also complement previous studies of fish diversity and biogeography in rivers of the Gulf and Atlantic slopes in the southeastern United States. As such, this group adds to the multiple other groups of aquatic organisms in this region for future comparative biogeographic analyses, but only if different clades that are being compared are of the same ages of divergences as determined by time-tree analyses or known abiotic factors. Comparisons of relationships in clades that diverged at different times conflate the comparative analysis and will likely lead to conflicting relationships with unknown reasons. Comparative time analyses are thus critical in future biogeographic studies of this group and others.

The phylogeographic patterns observed in species of* Pteronotropis *derived from phylogenetic analysis are largely consistent with previous studies of freshwater taxa inhabiting rivers occupied by members of* Pteronotropis*. The* P. hypselopterus* complex is widespread across the Gulf and Atlantic slopes and was once considered a single species. The status of this species changed with the elevation and descriptions of species (Suttkus and Mettee [7]) within the complex. Genetic data and analyses presented herein support the recognition of the species within this complex, as well as the need to recognize additional species, with the possible exception of the* P. merlini*, “*P. hypselopterus*” clade. These taxa will also likely possess additional diversity if examined more closely for morphological and genetic variation, as well as coloration of live breeding adults.

The southeastern fish fauna is second only to the Mississippi River drainage in terms of species diversity [21] and the challenge for taxonomists and systematists is to find and describe diversity before the extirpation of populations and species from these drainages that have been in isolation for millions of years. The detailed resolution and understanding of the phylogeography of species of* Pteronotropis* provide insights into the historical and contemporary events that were instrumental in the diversification of this group and offer insights into the importance of more dense sampling of any widespread taxa for clarity in diversification rates and patterns in a region.

## Figures and Tables

**(a):** 

Species drainage		Locality		Catalogue number	Extraction number	GenBank number
*Pteronotropis welaka *						
Cahaba R.	Lighseys pond	Bibb	AL	UAIC 10391	10	KP101134
Cahaba R.	Lightseys pond	Bibb	AL	UAIC 10391	11	KP101135
Apalachicola R.	Spring Cr.	Miller	GA	STL 1114.03	81	KP101136
Apalachicola R.	Spring Cr.	Miller	GA	STL 1114.03	82	KP101137
Choctawhatchee R.	Hathaway Mill	Holmes	FL	UAIC 14327.02	210	KP101138
Choctawhatchee R.	Hathaway Mill	Holmes	FL	UAIC 14327.02	211	KP101139
Choctawhatchee R.	Hathaway Mill	Holmes	FL	UAIC 14327.02	212	KP101140
*Pteronotropis harperi *						
Escambia R.	Hunter Cr.	Conecuh	AL	STL 862.01	65	KP101141
Escambia R.	Hunter Cr.	Conecuh	AL	STL 862.01	66	KM363660
Escambia R.	Patsaliga Cr.	Conecuh	AL	STL 367.01	67	KM363661
Escambia R.	Patsaliga Cr.	Conecuh	AL	STL 367.01	68	KM363662
Escambia R.	Patsaliga Cr.	Conecuh	AL	STL 367.01	69	KM363663
Chattahoochee R.	Kirkland Cr.	Early	GA	STL 689.03	70	KP101142
Chattahoochee R.	Kirkland Cr.	Early	GA	STL 689.03	71	KM363664
Apalachicola R.	Coolewahee Cr.	Baker	GA	STL 691.01	73	KM363665
Apalachicola R.	Coolewahee Cr.	Baker	GA	STL 691.01	74	KM363666
Apalachicola R.	Coolewahee Cr.	Baker	GA	STL 691.01	75	KM363667
Apalachicola R.	Spring Cr.	Miller	GA	STL 1114.01	83	KM363671
Apalachicola R.	Spring Cr.	Miller	GA	STL 1114.01	85	KM363672
*Pteronotropis hubbsi *						
Ouachita R.	Backwater pond	Ouachita	LA	UAIC 11928.01	06	KM363617
Ouachita R.	Backwater pond	Ouachita	LA	UAIC 11928.01	07	KM363617
Little R.	Little R.	McCurtin	OK	UAIC 12053	41	KM363643
Little R.	Little R.	McCurtin	OK	UAIC 12053	42	KM363644
*Pteronotropis grandipinnis *						
Apalachicola R.	Irwin Mill Cr.	Houston	AL	No voucher	12	KM363620
Apalachicola R,	Irwin Mill Cr.	Houston	AL	No voucher	13	KM363621
Apalachicola R,	Spring Cr.	Miller	GA	STL 1114.02	77	KM363668
Apalachicola R,	Spring Cr.	Miller	GA	STL 1114.02	78	KM363669
Apalachicola R.	Spring Cr.	Miller	GA	STL 1114.02	79	KM363670
Apalachicola R.	Beaver Cr.	Taylor	GA	STL 1129.01	116	KM363689
Apalachicola R.	Beaver Cr.	Taylor	GA	STL 1129.01	117	KM363690
Apalachicola R.	Cherokee Cr.	Lee	GA	GMNHTC 6252	141	KM363692
Apalachicola R.	Cherokee Cr.	Lee	GA	GMNHTC 6252	144	KP101143
*Pteronotropis hypselopterus *						
Mobile R.	Cedar Cr.	Mobile	AL	UAIC 12730.02	01	KM363612
Mobile R.	Cedar Cr.	Mobile	AL	UAIC 12730.02	02	KM363613
Mobile R.	Cedar Cr.	Mobile	AL	UAIC 12730.02	03	KM363614
Alabama R.	Little Reedy Cr.	Clarke	AL	UAIC 10850	18	KM363626
Yellow R.	Crooked Cr.	Covington	AL	UAIC 11026	19	KM363627
Choctawhatchee R.	Ponce DeLeon	Holmes	FL	UAIC 12649	20	KM363628
Yellow R.	Pond Cr.	Okaloosa	FL	UAIC 12594	25	KM363632
Escambia R.	Tenmile Cr.	Santa Rosa	FL	UAIC 12593	33	KM363636
Escambia R.	Tenmile Cr.	Santa Rosa	FL	UAIC 12593	34	KP101144
Tombigbee R.	Mill Cr.	Clarke	AL	UAIC 11050	38	KM363640
Tombigbee R.	Mill Cr.	Clarke	AL	UAIC 11050	39	KM363641
Tombigbee R.	Mill Cr.	Clarke	AL	UAIC 11050	40	KM363642
Escambia R.	Pritchett Mill Br.,	Escambia	FL	STL 684.03	55	KM363652
Choctawhatchee Bay	Garnier Cr.	Okaloosa	FL	STL 620.01	56	KM363653
Choctawhatchee Bay	Garnier Cr.	Okaloosa	FL	STL 620.01	57	KM363654
Choctawhatchee Bay	Garnier Cr.	Okaloosa	FL	STL 620.01	58	KM363655
Yellow R.	Turkey Hen Cr.	Okaloosa	FL	STL 685.02	59	KM363656
Yellow R.	Turkey Hen Cr.	Okaloosa	FL	STL 685.02	60	KM363657
Mobile Bay	Olive Cr.	Baldwin	AL	STL 363.02	62	KM363658
Blackwater R.	Blackwater R.	Okaloosa	FL	No voucher	161	KM363697
Blackwater R.	Blackwater R.	Okaloosa	FL	No voucher	162	KM363698
Blackwater R.	Blackwater R.	Okaloosa	FL	No voucher	163	KM363699
Blackwater R.	Ates Cr.	Santa Rosa	FL	No voucher	164	KM363700
Blackwater R.	Ates Cr.	Santa Rosa	FL	No voucher	165	KM363701
Blackwater R.	Ates Cr.	Santa Rosa	FL	No voucher	166	KM363702
Fish R.	Unnamed trib.	Baldwin	AL	UAIC 14317.01	167	KM363703
Fish R.	Unnamed trib.	Baldwin	AL	UAIC 14317.01	169	KM363704
Perdido R.	Blackwater R.	Baldwin	AL	UAIC 14318	173	KM363707
Perdido R.	Blackwater R.	Baldwin	AL	UAIC 14318	174	KM363708
Perdido R.	Blackwater R.	Baldwin	AL	UAIC 14318	175	KM363709
Escambia R.	Pine Barren Cr	Escambia	FL	UAIC 14320	179	KM363710
Blackwater R.	Cobb Cr.	Santa-Rosa	FL	UAIC 14321	182	KM363711
Blackwater R.	Cobb Cr.	Santa-Rosa	FL	UAIC 14321	183	KM363712
Blackwater R.	Cobb Cr.	Santa-Rosa	FL	UAIC 14321	184	KM363713
Blackwater R.	Ates Cr.	Santa-Rosa	FL	UAIC 14322.01	185	KM363714
Yellow R.	Julian Mill Cr.	Santa-Rosa	FL	UAIC 14323.01	191	KM363715
Yellow R.	Julian Mill Cr.	Santa-Rosa	FL	UAIC 14323.01	192	KM363716
Yellow R.	Julian Mill Cr.	Santa-Rosa	FL	UAIC 14323.01	193	KM363717
Yellow R.	Juniper Cr.	Okaloosa	FL	UAIC 14324	195	KM363718
Yellow R.	Juniper Cr.	Okaloosa	FL	UAIC 14324	196	KM363719
Yellow R.	Juniper Cr.	Okaloosa	FL	UAIC 14324	197	KM363720
Yellow R.	Mill Cr.	Okaloosa	FL	UAIC 14325.01	198	KM363721
Yellow R.	Mill Cr.	Okaloosa	FL	UAIC 14325.01	199	KM363722
Yellow R.	Mill Cr.	Okaloosa	FL	UAIC 14325.01	200	KM363723
Choctawhatchee R.	Blue Cr.	Holmes	FL	UAIC 14326	204	KM363724
Choctawhatchee R.	Blue Cr.	Holmes	FL	UAIC 14326	205	KM363725
Choctawhatchee R.	Blue Cr.	Holmes	FL	UAIC 14326	206	KM363726
Choctawhatchee R.	Hathaway Mill	Holmes	FL	UAIC 14327.01	207	KM363727
Choctawhatchee R.	Hathaway Mill	Holmes	FL	UAIC 14327.01	208	KM363728
Choctawhatchee R.	Hathaway Mill	Holmes	FL	UAIC 14327.01	209	KM363729
Choctawhatchee R.	Wrights Cr.	Holmes	FL	UAIC 14328.01	213	KM363730
Choctawhatchee R.	Wrights Cr.	Holmes	FL	UAIC 14328.01	214	KM363731
Choctawhatchee R.	Seven Runs Cr.	Holmes	FL	UAIC 14329.01	217	KM363732
Choctawhatchee R.	Seven Runs Cr.	Holmes	FL	UAIC 14329.01	218	KM363733
St. Andrews Bay	Cooks Bayou	Bay	FL	UAIC 14330	219	KM363734
St. Andrews Bay	Cooks Bayou	Bay	FL	UAIC 14330	220	KM363735
St. Andrews Bay	Cooks Bayou	Bay	FL	UAIC 14330	221	KM363736
St. Andrews Bay	Unnamed trib.	Bay	FL	UAIC 14331	222	KM363737
St. Andrews Bay	Unnamed trib.	Bay	FL	UAIC 14331	223	KM363738
St. Andrews Bay	Unnamed trib.	Bay	FL	UAIC 14331	224	KM363739
Choctawhatchee Bay	Bear Cr.	Bay	FL	UAIC 14332	225	KM363740
Choctawhatchee Bay	Bear Cr.	Bay	FL	UAIC 14332	226	KM363741
Choctawhatchee R.	Spring Cr.	Geneva	AL	UAIC 14343	255	KM363756
Choctawhatchee R.	Spring Cr.	Geneva	AL	UAIC 14343	256	KM363757
Choctawhatchee R.	Spring Cr.	Geneva	AL	UAIC 14343	257	KM363758
*Pteronotropis* sp. cf. *hypselopterus *						
St. Johns R.	Little Orange Cr.,	Putnam	FL	UAIC 12290	28	KP101145
St. Johns R.	Juniper Cr.,	Marion	FL	GMNH5380	149	KP101146
St. Johns R.	Juniper Cr.	Marion	FL	GMNH5380	150	KP101147
St. Johns R.	Juniper Cr.	Marion	FL	GMNH5380	151	KP101148
Alafia R.	Hurrah Cr.	Hillsborough	FL	UAIC 14339	243	KP101149
Alafia R.	Hurrah Cr.	Hillsborough	FL	UAIC 14339	244	KP101150
Alafia R.	Hurrah Cr.	Hillsborough	FL	UAIC 14339	245	KP101151
St. Johns R.	Juniper Cr.	Marion	FL	UAIC 14340	246	KP101152
St. Johns R.	Juniper Cr.	Marion	FL	UAIC 14340	247	KP101153
St. Johns R.	Juniper Cr.	Marion	FL	UAIC 14340	248	KP101154
St. Johns R.	Alexander Spr.	Lake	FL	UAIC 14341	249	KP101155
St. Johns R.	Alexander Spr.	Lake	FL	UAIC 14341	250	KP101156
St. Johns R.	Alexander Spr.	Lake	FL	UAIC 14341	251	KP101157
*Pteronotropis euryzonus *						
Chattahoochee R.	Maringo Cr.	Russell	AL	UAIC 12229	22	KM363629
Chattahoochee R.	Snake Cr.	Russell	AL	UAIC 10493	51	KM363648
Chattahoochee R.	Snake Cr.	Russell	AL	UAIC 10493	52	KM363649
Chattahoochee R.	Snake Cr.	Russell	AL	UAIC 10493	53	KM363650
Chattahoochee R.	Snake Cr.	Russell	AL	UAIC 10493	54	KM363651
Chattahoochee R.	Uchee Cr.	Russell	AL	UAIC 14344	258	KM363759
Chattahoochee R.	Uchee Cr.	Russell	AL	UAIC 14344	259	KM363760
Chattahoochee R.	Uchee Cr.	Russell	AL	UAIC 14344	260	KM363761
*Pteronotropis merlini *						
Choctawhatchee R.	Claybank Cr.	Dale	AL	UAIC 12595	08	KM363617
Choctawhatchee R.	Claybank Cr.	Dale	AL	UAIC 12595	09	KM363619
Pea R.	Clearwater Cr.	Coffee	AL	No voucher	15	KM363623
Pea R.	Clearwater Cr.	Coffee	AL	No voucher	16	KM363624
Pea R.	Clearwater Cr.	Coffee	AL	No voucher	17	KM363625
Choctawhatchee R.	W. Fork	Barbour	AL	UAIC 12735	29	KM363633
Choctawhatchee R.	W. Fork	Barbour	AL	UAIC 12735	30	KM363634
Choctawhatchee R.	W. Fork	Barbour	AL	UAIC 12735	31	KM363635
Choctawhatchee R.	Unnamed trib.	Geneva	AL	UAIC 14342	252	KM363754
Choctawhatchee R.	Unnamed trib.	Geneva	AL	UAIC 14342	254	KM363755
Choctawhatchee R.	Unnamed trib.	Geneva	AL	UAIC 14342	261	KP101158
*Pteronotropis metallicus *						
Suwannee R.	Sampson R.	Bradfrod	FL	UF 158855	96	KM363673
Ochlockonee R.	Rocky Comf. Cr.	Gadsden	FL	No voucher	155	KM363693
Ochlockonee R.	Rocky Comf. Cr.	Gadsden	FL	No voucher	156	KM363694
Ochlockonee R.	Rocky Comf. Cr.	Gadsden	FL	No voucher	157	KM363695
St. Marks R.	St. Marks R.	Leon	FL	No voucher	159	KM363696
St. Marks R.	Chicken Br.	Leon	FL	UAIC 14334	231	KM363742
St. Marks R.	Chicken Br.	Leon	FL	UAIC 14334	232	KM363743
St. Marks R.	Chicken Br.	Leon	FL	UAIC 14334	233	KM363744
Suwannee R.	Hunter Cr.	Hamilton	FL	UAIC 14336	234	KM363745
Suwannee R.	Hunter Cr.	Hamilton	FL	UAIC 14336	235	KM363746
Suwannee R.	Hunter Cr.	Hamilton	FL	UAIC 14336	236	KM363747
St. Marys R.	Cedar Cr.	Baker	FL	UAIC 14337	237	KM363748
St. Marys R.	Cedar Cr.	Baker	FL	UAIC 14337	238	KM363749
St. Marys R.	Cedar Cr.	Baker	FL	UAIC 14337	239	KM363750
Suwannee R.	Santa-Fe R.	Gilchrist	FL	UAIC 14338	240	KM363751
Suwannee R.	Santa-Fe R.	Gilchrist	FL	UAIC 14338	241	KM363752
Suwannee R.	Santa-Fe R.	Gilchrist	FL	UAIC 14338	242	KM363753
*Pteronotropis signipinnis *						
Pascagoula R.	Beaverdam Cr.	Forest	MS	UAIC 13416.03	04	KM363615
Pascagoula R.	Beaverdam Cr.	Forest	MS	UAIC 13416.03	05	KM363616
Mobile R.	Cedar Cr.	Mobile	AL	UAIC 12730.15	23	KM363630
Mobile R.	Cedar Cr.	Mobile	AL	UAIC 12730.15	24	KM363631
Tensaw R.	Ferris Cr.	Baldwin	AL	UAIC 11056	35	KM363637
Tensaw R.	Ferris Cr.	Baldwin	AL	UAIC 11056	36	KM363638
Tensaw R.	Ferris Cr.	Baldwin	AL	UAIC 11056	37	KM363639
Pearl R.	Lawrence Cr.	Washington	LA	UAIC 12204	44	KM363645
Pearl R.	Lawrence Cr.	Washington	LA	UAIC 12204	45	KM363646
Pearl R.	Lawrence Cr.	Washington	LA	UAIC 12204	46	KM363647
Biloxi R.	Saucier Cr.	Harrison	MS	STL 85563	63	KM363659
Fish R.	Unnamed trib.	Baldwin	AL	UAIC 14317.02	171	KM363705
Fish R.	Unnamed trib.	Baldwin	AL	UAIC 14317.02	172	KM363706
*Pteronotropis *sp. cf.* signipinnis *						
Escambia R.	Pritchett Mill	Escambia	FL	UAIC 684.02	64	KP101159
Perdido R.	Beartree Cr.	Baldwin	AL	UAIC 14319	177	KP101160
Perdido R.	Beartree Cr.	Baldwin	AL	UAIC 14319	178	KP101161
Yellow R.	Julian Mill Cr.	Santa-Rosa	FL	UAIC 14323.02	194	KP101162
Yellow R.	Mill Cr.	Okaloosa	FL	UAIC 14325.02	201	KP101163
Yellow R.	Mill Cr.	Okaloosa	FL	UAIC 14325.02	202	KP101164
Yellow R.	Mill Cr.	Okaloosa	FL	UAIC 14325.02	203	KP101165
Apalachicola R.	Fourmile Cr.	Calhoun	FL	UAIC 14333	228	KP101166
Apalachicola R.	Fourmile Cr.	Calhoun	FL	UAIC 14333	229	KP101167
Apalachicola R.	Fourmile Cr.	Calhoun	FL	UAIC 14333	230	KP101168
*Pteronotropis stonei *						
Santee R.	Jacks Cr.	Clarendon	SC	UAIC 12590	14	KM363622
Santee R.	Unnamed Cr.	Calhoun	SC	STL 1120.01	98	KM363674
Santee R.	Unnamed Cr.	Calhoun	SC	STL 1120.01	99	KM363675
N. Fork Edisto R.	Murphy Mill Cr.	Calhoun	SC	STL 1121.01	100	KM363676
N. Fork Edisto R.	Murphy Mill Cr.	Calhound	SC	STL 1121.01	101	KM363677
Combahee R.	Savannah Cr.	Colleton	SC	STL 1122.01	102	KM363678
Combahee R.	Savannah Cr.	Colleton	SC	STL 1122.01	103	KM363679
Combahee R.	Salkehatchie R.	Barnwell	SC	STL 1123.01	105	KM363680
Santee R.	Congaree Cr.	Lexington	SC	STL 1124.01	106	KM363681
Santee R.	Congaree Cr.	Lexington	SC	STL 1124.01	107	KM363682
N. Fork Edisto R.	Black Cr.	Lexington	SC	STL 1125.01	108	KM363683
N. Fork Edisto R.	Black Cr.	Lexington	SC	STL 1125.01	109	KP101169
Savannah R.	Cedar Cr.	Aiken	SC	STL 1126.01	110	KP101171
Savannah R.	Cedar Cr.	Aiken	SC	STL 1126.01	111	KM363684
S. Fork Edisto R.	Unnamed trib.	Aiken	SC	STL 1127.01	112	KM363685
S. Fork Edisto R.	Unnamed trib.	Aiken	SC	STL 1127.01	113	KM363686
Savannah R.	Boggy Gut Cr.	Richmond	GA	STL 1128.01	114	KM363687
Savannah R.	Boggy Gut Cr.	Richmond	GA	STL 1128.01	115	KM363688
Pee Dee R.	Beaver Dam Cr.	Kershaw	SC	STL 1130.01	118	KM363691
Pee Dee R.	Beaver Dam Cr.	Kershaw	SC	STL 1130.01	119	KP101171

**(b):** 

Outgroup taxa			
Species	GenBank number	Species	GenBank number
*Cyprinella labrosa *	AF111258.1	*Cyprinella lutrensis *	AF111210
* *		* *	AF111210.11
*Cyprinella monacha *	AF111228.1	*Cyprinella zanema *	AF111230.1
*Lythrurus roseipinnis *	AF111231.1	*Notropis atherinoides *	AF111232.1
*Notropis baileyi *	EF613593.1	*Notropis stramineus *	NC 008110.1
*Notropis texanus *	EF613581.1		

**Table 2:** Statistical values for *Pteronotropis signipinnis*, *P*. *euryzonus*, and the *P*. *hypselopterus* complex.

Clade	*N*	*H*	*H*_D_	*π*	*D*	*P* value
*P*. *hypselopterus *	40	29	0.858	0.042	−1.330	>.05
*P*. *signipinnis *	23	14	0.881	0.066	2.500	<.05^*^
*P*. *merlini *	30	16	0.874	0.008	0.465	>.05
*P*. *grandipinnis *	17	11	0.962	0.014	0.221	>.05
*P*. *stonei *	20	10	0.943	0.031	1.002	>.05
*P*. *euryzonus *	8	3	0.464	0.000	−1.448	>.05
*P*. *metallicus *	32	27	0.994	0.058	−0.483	>.05
*P*. *signipinnis *eastern	10	9	0.978	0.007	−0.071	>.05
*P*. *signipinnis *western	13	5	0.628	0.003	−1.882	<.05^*^
*P*. *signipinnis* Ochlockonee	19	16	0.989	0.059	−0.521	>.05
*P. signipinnis *St. Johns	13	11	0.987	0.010	−0.060	>.05
*P*. *hypselopterus *eastern	27	20	0.872	0.107	−1.23	>.05
*P*. *hypselopterus *western	13	9	0.723	0.002	−1.17	>.05

*N*: number of individuals, *H*: haplotype number, *H*_D_: haplotype diversity, *π*: nucleotide diversity, *D*: Tajamas *D* statistic, and *P* value: the *P* value associated with Tajamas *D* statistic (^*^indicating a significant value).

**Table 3:** Corrected percent divergence values with standard errors (above diagonal) and pairwise differences with standard errors (below diagonal) for the *Pteronotropis  hypselopterus* complex, *P*. *euryzonus,* and *P*. *signipinnis* for ND2.

Clade		1	2	3	4	5	6	7
1	*P*. *hypselopterus *	**4.8 ± 1.2**	22.6 ± 4.5	8.2 ± 2.2	9.4 ± 2.5	15.3 ± 3.6	10.4 ± 3.2	11.8 ± 2.6
2	*P*. *signipinnis *	17.4 ± 2.7	**10.7 ± 2.6**	18.4 ± 2.8	18.3 ± 2.8	19.5 ± 2.8	18.1 ± 2.8	18.0 ± 2.5
3	*P*. *merlini *	7.0 ± 1.6	18.4 ± 2.8	**1.6 ± 0.7**	8.5 ± 2.2	10.3 ± 2.2	8.5 ± 2.1	9.2 ± 2.5
4	*P*. *grandipinnis *	8.2 ± 2.1	18.3 ± 2.8	8.5 ± 2.2	**0.7 ± 0.4**	10.6 ± 2.3	6.4 ± 1.9	10.2 ± 2.3
5	*P*. *stonei *	12.4 ± 2.4	19.5 ± 2.8	10.3 ± 2.2	10.6 ± 2.3	**03.3 ± 1.0**	10.1 ± 2.2	9.6 ± 1.8
6	*P*. *euryzonus *	8.5 ± 2.0	18.1 ± 2.8	8.5 ± 2.1	6.4 ± 1.9	10.0 ± 2.2	**0.0 ± 0.0**	9.0 ± 1.9
7	*P*. *metallicus *	9.8 ± 1.9	18.0 ± 1.9	9.2 ± 1.9	10.2 ± 2.3	9.6 ± 1.8	9.0 ± 1.9	**7.7 ± 2.0**

Bold face values indicate within group variation.
